# Recovered Glomerular Counts in Relation to Kidney Mass and Developmental Transcriptomes in FVB/N Mice

**DOI:** 10.3390/ijms272110056

**Published:** 2026-11-09

**Authors:** Tomoki Omori, Masaki Watanabe, Ryuya Nakagawa, Takeru Sasaki, Nobuya Sasaki

**Affiliations:** Laboratory of Laboratory Animal Science and Medicine, School of Veterinary Medicine, Kitasato University, 35-1, Higashi-23, Towada 034-8628, Aomori, Japanwatanabe.masaki@kitasato-u.ac.jp (M.W.);

**Keywords:** nephron endowment, inbred mouse strains, FVB/N, reference genome bias, kidney development, glomerular long-axis diameter, albuminuria, RNA sequencing

## Abstract

Low nephron endowment is associated with hypertension and chronic kidney disease. We compared recovered glomerular counts and kidney mass across four mouse strains at 8 weeks of age and examined age-associated phenotypes and developmental kidney transcriptomes in FVB/N and C57BL/6J mice. Sex-adjusted models allowing for strain-specific variances showed lower recovered counts and counts per gram of kidney in FVB/N than in the other strains. Proportional scaling with kidney mass was not supported. After adjustment for kidney weight, differences remained significant relative to BALB/c and DBA/2, whereas the difference from C57BL/6J remained unresolved. These measurements do not establish absolute nephron endowment. In independent groups of males of different ages, FVB/N mice showed greater glomerular enlargement and higher semiquantitative urinary albumin-to-creatinine ratios. Strain-matched developmental RNA-seq attenuated measurable reference-genome effects. Deconvolution using either the P1 or E14.5 single-cell reference detected no strain difference in the estimated progenitor proportion at E13.5, although absolute estimates were reference-dependent. No coordinated GDNF–RET downregulation or significant kidney-related enrichment was detected. Integration of regulatory annotations prioritised five genes as exploratory candidates. Lower interferon-related expression in FVB/N at P1 was consistent across samples and leave-one-out analyses. Developmental phenotyping and independent genetic validation are needed to establish the origins of these strain differences.

## 1. Introduction

Nephron number is fixed by term birth in humans [1] and by approximately postnatal day 3–4 in mice [2,3], and varies several-fold among individuals: a stereological survey of human kidneys at autopsy reported an almost nine-fold range, from 210,332 to 1,825,380 glomeruli [4]. Low nephron endowment is associated with hypertension and susceptibility to chronic kidney disease, an association usually explained by hyperfiltration in the remaining nephrons and subsequent glomerular hypertrophy and sclerosis [5,6,7]. Identifying the determinants of nephron endowment is therefore relevant to both clinical and developmental research.

Inbred mouse strains provide a tractable system for investigating nephron endowment because renal phenotypes vary among strains while animals within a strain are genetically similar. Strain differences have been reported in glomerular location [8] and in susceptibility to experimental nephropathy, including diabetic models [9] and adriamycin-induced injury [10,11]. In the latter, disease initiation is governed largely by a missense variant in *Prkdc*, whereas severity is modified by genetic background [10,11]. Total nephron number has been compared between C3H/HeJ and C57BL/6J mice [12]. Long et al. reported fewer glomeruli in FVB/N than in sex-matched B6 mice at 18 weeks, both per kidney and after normalisation to kidney or body weight, together with higher albuminuria [13]. The association of the FVB/N background with lower glomerular counts and albuminuria was therefore established before the present study.

Reduced nephron endowment may increase the filtration load on the remaining glomeruli, leading to compensatory enlargement and, over time, proteinuria [5,6,7]. Age-associated glomerular and urinary phenotypes in uninjured kidneys can therefore complement baseline strain comparisons, while remaining distinct from susceptibility to induced renal injury [10,11].

Comparing developmental transcriptomes between strains may help identify the basis of these differences, but reference-genome bias complicates this approach. The mouse reference genome GRCm39 is derived from C57BL/6J; reads from other strains therefore contain more mismatches and may align or be assigned to genes less efficiently. This bias is often greatest in highly divergent genes, which may then be preferentially selected as candidates. In analyses based on a single reference genome, technical mapping bias can be confounded with biological expression differences. Strain-specific genomes or pseudogenomes constructed from known variants can reduce this problem [14,15,16], but few developmental studies have quantified both the bias and the effect of correction.

We combined a baseline comparison of four strains with an assessment of age-associated phenotypes in males and strain-specific analyses of developmental transcriptomes. First, we analysed recovered glomerular counts and their relationship to kidney mass. Second, we compared glomerular long-axis diameter and semiquantitative UACR estimates in independent groups of male C57BL/6J and FVB/N mice of different ages. Third, we examined E13.5 and P1 kidney transcriptomes using strain-matched quantification, sensitivity analyses with different single-cell references and nephrogenic gene sets. We integrated variant, chromatin and expression data to prioritise candidates for independent testing.

## 2. Results

### 2.1. Recovered Counts and Their Relation to Kidney Mass

**Figure 1:**
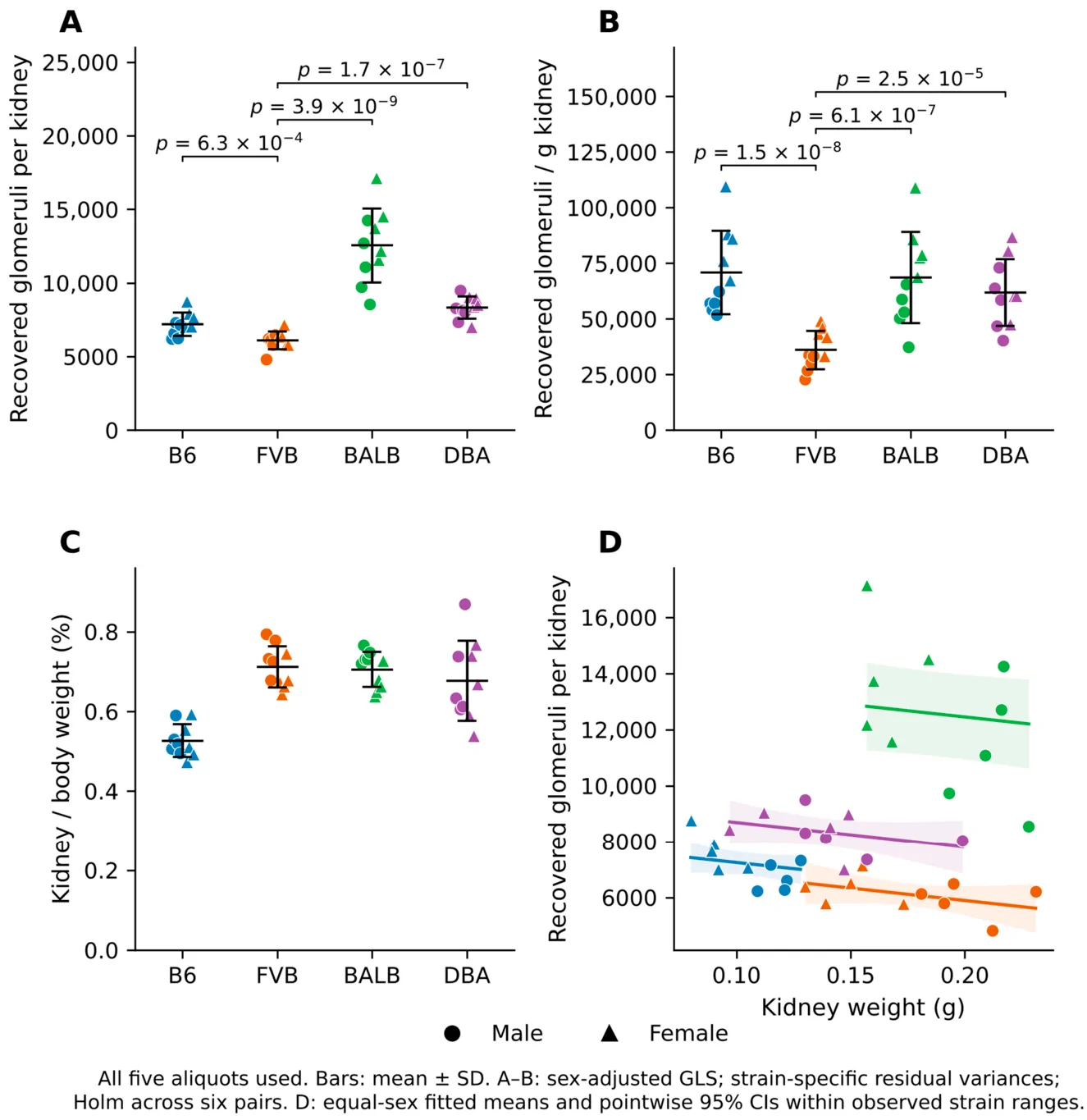
Recovered glomerular counts and kidney mass at 8 weeks. (**A**) Recovered counts per kidney. (**B**) Recovered glomerular count per gram of kidney. (**C**) Kidney-to-body-weight ratio. (**D**) Recovered count versus kidney weight with fitted means from the additive sex- and kidney-weight-adjusted GLS model. Circles and triangles indicate males and females, respectively (5 of each per strain). Bars in (**A**–**C**) show mean ± SD. *p* values in (**A**,**B**) were obtained from sex-adjusted GLS models with strain-specific residual variances and Holm correction across all six strain pairs. Lines in (**D**) represent fitted means standardised to equal representation of both sexes and pointwise 95% CIs, shown only over observed strain-specific kidney-weight ranges. Recovered counts do not establish absolute nephron endowment. B6, C57BL/6J (blue); FVB, FVB/N (orange); BALB, BALB/c (green); DBA, DBA/2 (purple).

Glomeruli recovered after isolation were counted in four inbred strains at 8 weeks of age (*n* = 5 males and 5 females per strain; Table 1, Figure 1).

All models used for strain comparisons included sex and allowed a separate residual variance for each strain. No strain × sex interaction was detected (Wald *p* = 0.294). Pairwise contrasts and confidence intervals are provided in Supplementary Table S4.

Mean recovered counts were 7196 ± 778 in C57BL/6J, 6104 ± 614 in FVB/N, 12,548 ± 2506 in BALB/c and 8330 ± 758 in DBA/2 (Figure 1A). FVB/N had lower recovered counts than C57BL/6J (difference −1092; 95% CI, −1646 to −538; Holm-adjusted *p* = 0.00063), BALB/c and DBA/2 (both adjusted *p* < 0.001).

Recovered glomerular count per gram of kidney was also lower in FVB/N than in each of the other strains: 36,071 ± 8622 compared with 70,924 ± 18,727 in C57BL/6J, 68,592 ± 20,488 in BALB/c and 61,860 ± 14,947 in DBA/2 (all Holm-adjusted *p* < 0.001; Figure 1B). Kidney-to-body-weight ratios are shown in Figure 1C.

After kidney-weight adjustment, the FVB/N–C57BL/6J difference was −468 (95% CI, −1603 to +666; adjusted *p* = 0.407). Adjusted differences remained significant relative to BALB/c (−6561; 95% CI, −8123 to −4998) and DBA/2 (−1912; 95% CI, −2712 to −1111); both adjusted *p* < 0.001. Fitted relationships are shown in Figure 1D.

The log–log sensitivity model yielded the same pattern of statistical significance: the adjusted FVB/N-to-C57BL/6J count ratio was 0.967 (95% CI, 0.819–1.141; adjusted *p* = 0.680). The common allometric exponent was −0.256 (95% CI, −0.539 to +0.028), which differed significantly from the value of 1 required for proportional scaling (*p* < 0.001). Strain-by-kidney-weight interactions were not significant on the linear or log scale (Wald *p* = 0.269 and 0.520). Kidney-weight ranges did not overlap between C57BL/6J (0.080–0.128 g) and FVB/N (0.130–0.231 g).

Mean within-kidney technical CVs were 11.37%, 12.37%, 14.16% and 10.08% in C57BL/6J, FVB/N, BALB/c and DBA/2, respectively. A sex-adjusted analysis of log CV with HC3 inference did not detect an overall strain effect (*p* = 0.0663); no pairwise contrast was significant after Holm correction. Aliquot-level observations are supplied in Supplementary Data S1, with summaries in Supplementary Table S3.

### 2.2. FVB/N Shows Greater Age-Associated Glomerular Enlargement and Higher Semiquantitative UACR Estimates

**Figure 2:**
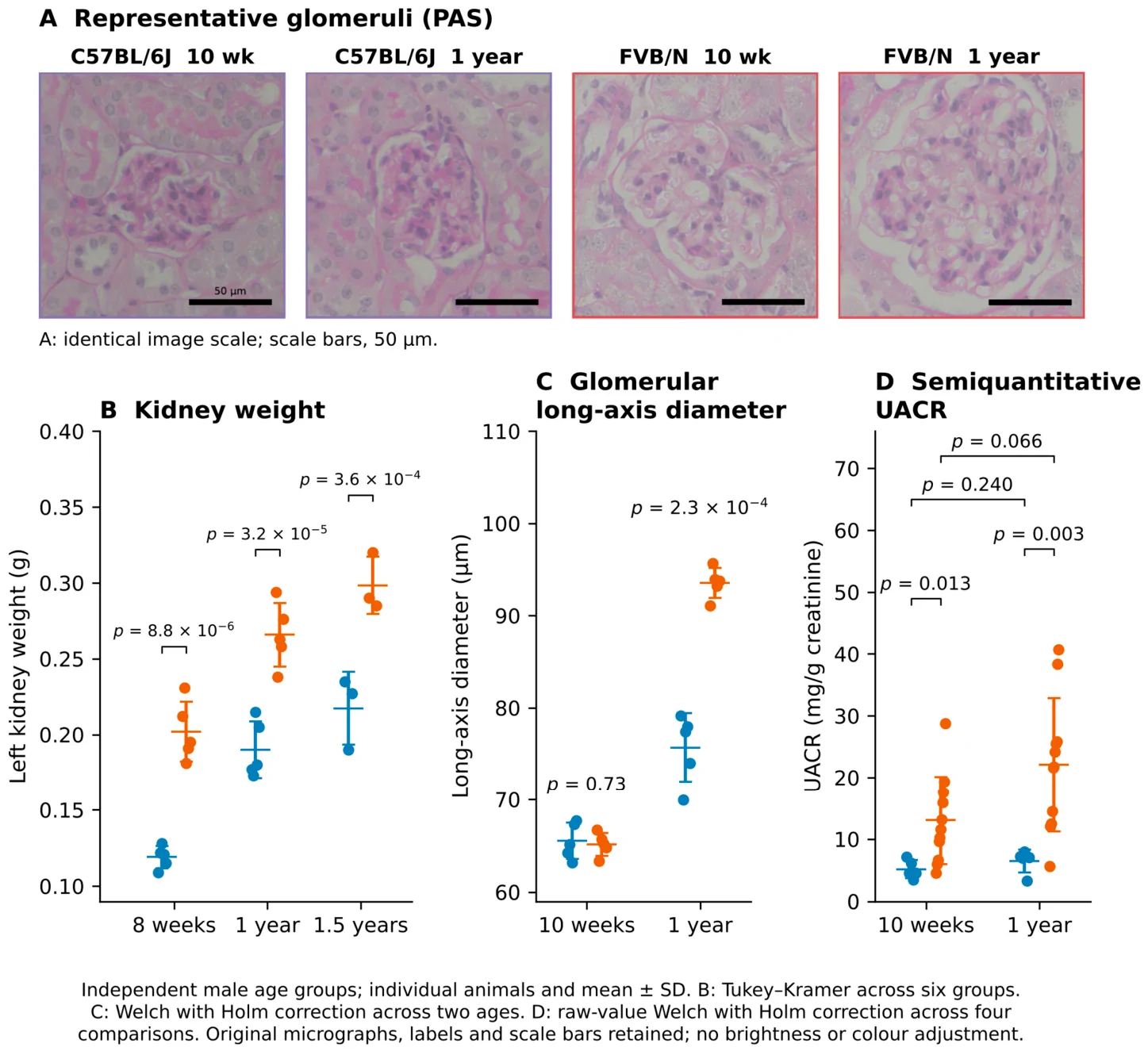
Age-associated renal and glomerular phenotypes in male C57BL/6J and FVB/N mice. (**A**) Representative PAS-stained superficial cortical glomeruli at 10 weeks and 1 year, at the same scale; scale bars, 50 µm. Original micrograph colours were retained. (**B**) Kidney weight at 8 weeks, 1 year and 1.5 years (*n* = 5, 5 and 3 per strain); 8-week animals are the same males as in Figure 1. (**C**) Per-animal mean glomerular long-axis diameter (20 glomeruli per animal; *n* = 5 per strain per age). (**D**) Semiquantitative UACR estimates (C57BL/6J *n* = 5, FVB/N *n* = 11 per age). Points represent individual animals, and error bars indicate SD; blue, C57BL/6J; orange, FVB/N. Age groups are independent. Panel (**B**) uses Tukey–Kramer comparisons across all six strain-by-age groups; panel (**C**) uses Welch tests with Holm correction across two ages. Panel (**D**) uses Welch tests on raw values with Holm correction applied to a single family comprising two between-strain and two within-strain age comparisons. The BSA-based UACR assay was not independently validated for band identity or linearity.

Age-associated differences in kidney mass, glomerular morphology and urinary estimates were assessed in male C57BL/6J and FVB/N mice (Figure 2).

Kidney mass increased with age in both strains (Figure 2B). Kidney weight rose from 0.119 ± 0.007 g at 8 weeks (*n* = 5) to 0.190 ± 0.019 g at 1 year (*n* = 5) and 0.217 ± 0.024 g at 1.5 years (*n* = 3) in C57BL/6J, and from 0.202 ± 0.020 g to 0.266 ± 0.021 g and 0.298 ± 0.019 g, respectively, in FVB/N (two-way ANOVA: strain F(1,20) = 122.0, *p* = 5.8 × 10^−10^; age F(2,20) = 61.1, *p* = 3.0 × 10^−9^; strain × age F(2,20) = 0.10, *p* = 0.91). FVB/N kidneys were heavier at every age examined (adjusted *p* = 8.8 × 10^−6^, 3.2 × 10^−5^ and 3.6 × 10^−4^ at 8 weeks, 1 year and 1.5 years, respectively), and there was no evidence that the between-strain difference widened with age.

Glomerular long-axis diameter increased more with age in FVB/N (Figure 2A,C). No difference was detected at 10 weeks (C57BL/6J, 65.5 ± 2.0 µm; FVB/N, 65.1 ± 1.2 µm; adjusted *p* = 0.73; *n* = 5 per strain), whereas FVB/N had a larger diameter at 1 year (93.5 ± 1.6 versus 75.7 ± 3.7 µm in C57BL/6J; adjusted *p* = 2.3 × 10^−^⁴). The cross-sectional increase in FVB/N was nearly three times that in C57BL/6J (+28.4 versus +10.2 µm; strain × age interaction F(1,16) = 75.4, *p* = 1.9 × 10^−7^).

Semiquantitative UACR estimates were higher in FVB/N at both ages after correction (Figure 2D): 13.0 ± 7.1 versus 5.2 ± 1.5 mg/g creatinine at 10 weeks (adjusted *p* = 0.013), and 22.0 ± 10.8 versus 6.5 ± 1.8 at 52 weeks (adjusted *p* = 0.0028). Within-strain age comparisons were not significant after correction (C57BL/6J, adjusted *p* = 0.240; FVB/N, adjusted *p* = 0.066). Welch tests on log-transformed values yielded the same pattern of statistical significance. The strain × age interaction for log-transformed UACR was not significant (*p* = 0.43).

### 2.3. Strain-Matched Quantification Changes Expression Estimates

Kidney RNA was sequenced at E13.5 and P1 (3 males per strain per stage; mean 29.0 million read pairs per sample; Supplementary Table S2). Sample-level PCA is provided in Supplementary Figure S2A–C. All 12 samples were retained, including FVBP1–3, which had a lower unique-mapping fraction.

**Figure 3:**
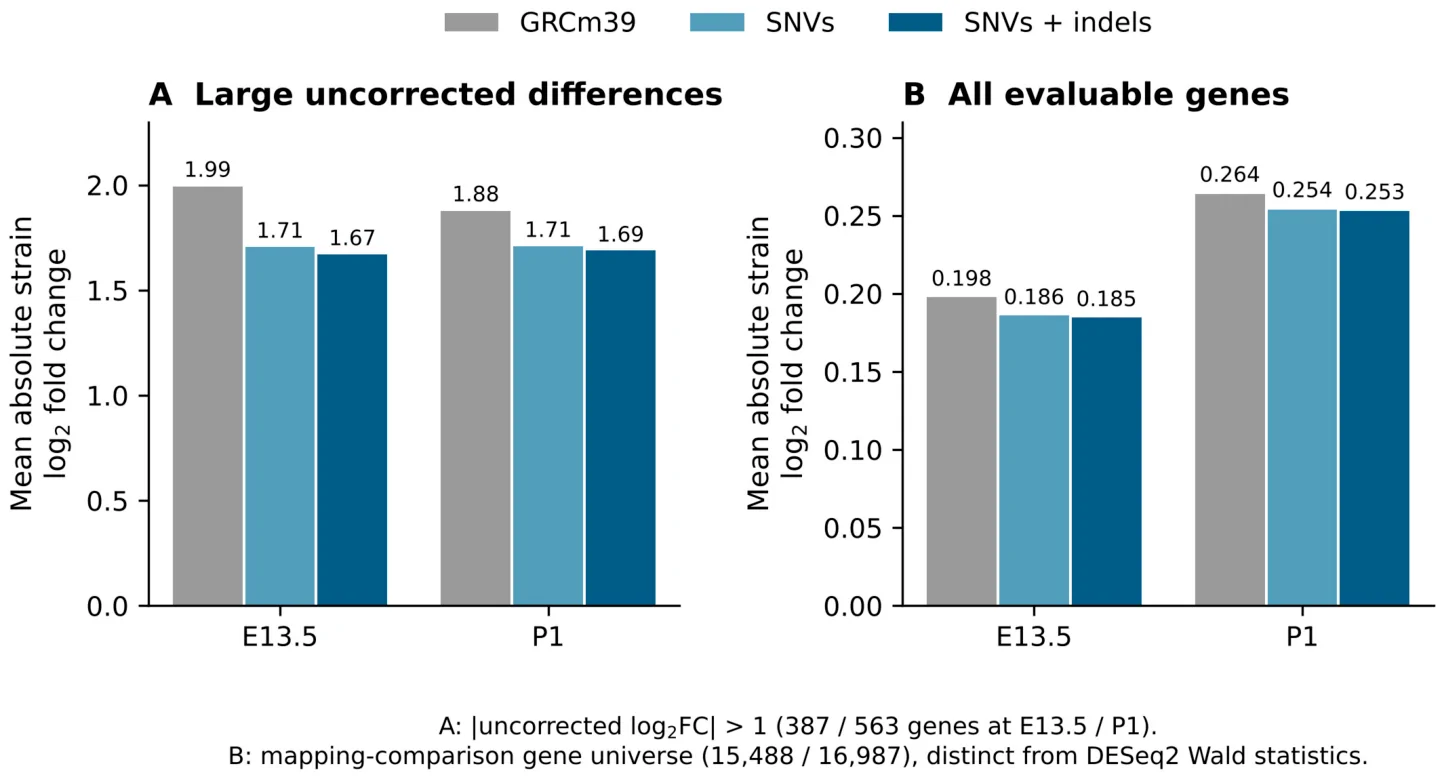
Effect of strain-specific quantification on expression estimates. Mean absolute strain log_2_ fold change using GRCm39, an SNV-only personalised genome and an SNV-plus-indel personalised genome, for (**A**) genes with |uncorrected log_2_FC| > 1 (387 at E13.5; 563 at P1) and (**B**) all genes in the mapping-comparison tables (15,488 and 16,987, respectively). These mapping-comparison summaries are distinct from DESeq2 Wald statistics. Detailed mapping and coordinate-conversion checks are provided in the Supplementary Materials.

The choice of reference genome altered estimates of expression differences, particularly among genes with large uncorrected strain differences. Mean absolute log_2_FC in this selected group fell from 1.99 to 1.67 at E13.5 and from 1.88 to 1.69 at P1 with SNV-plus-indel quantification (Figure 3A). The average changes across all evaluable genes were smaller (Figure 3B).

GRCm39-only quantification retained 1101 of 1186 differentially expressed genes at E13.5 and 1367 of 1441 at P1 (Jaccard indices 0.80 and 0.84). Genes classified as differentially expressed with only one quantification approach were concentrated among the reference-sensitive genes. The GDNF–RET, kidney gene-set, candidate and P1 interferon findings were retained. E13.5 translation- and ribosome-related sets were negatively enriched with GRCm39-only counts but had positive but nonsignificant enrichment scores with strain-matched counts (Supplementary Table S5; Supplementary Note S2).

### 2.4. Expression Differences Are Widespread, but the GDNF–RET Axis Does Not Show Coordinated Downregulation

**Figure 4:**
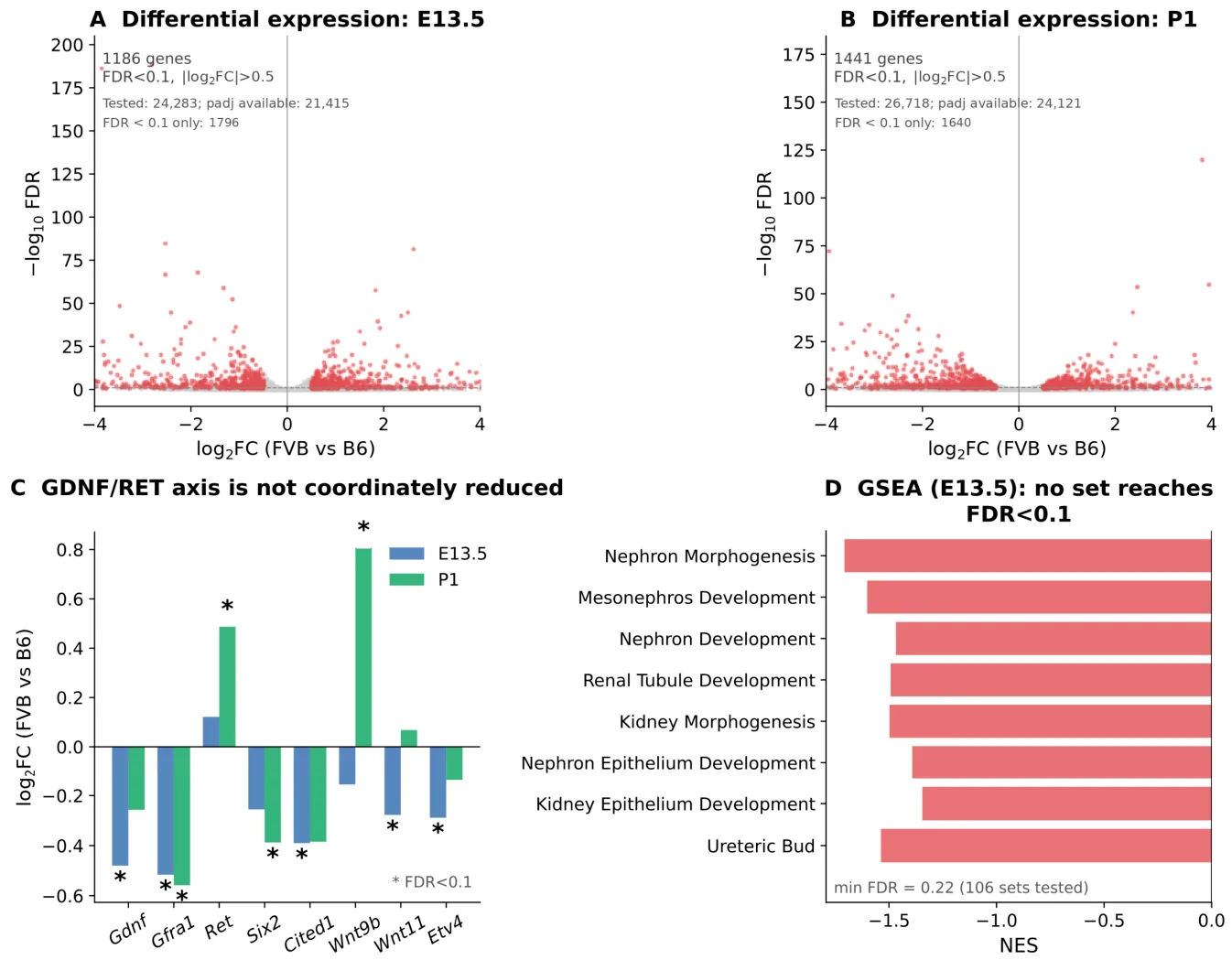
Developmental kidney expression differences between FVB/N and C57BL/6J. Volcano plots show differential expression at (**A**) E13.5 and (**B**) P1. Of 24,283 and 26,718 genes tested at E13.5 and P1, 21,415 and 24,121 had a nonmissing adjusted *p* value; 1796 and 1640 reached FDR < 0.1. The gene counts shown in panels A and B additionally apply the criterion |log_2_FC| > 0.5: 1186 genes at E13.5 and 1441 at P1. Red points meet both criteria and grey points do not; the dashed horizontal line marks FDR = 0.1, and the vertical line marks log_2_FC = 0. (**C**) Log_2_ fold changes in genes of the GDNF–RET axis; asterisks indicate FDR < 0.1; blue and green bars denote E13.5 and P1, respectively. *Six2* was significantly lower at P1 (log_2_FC −0.39). (**D**) Gene-set enrichment for 106 kidney-related gene sets at E13.5, ordered by adjusted *p* value. No set reached FDR < 0.1; the minimum adjusted *p* value was 0.22. The corresponding minimum at P1 was 0.35. Fold changes are FVB/N versus C57BL/6J.

At FDR < 0.1 and |log_2_FC| > 0.5, 1186 genes at E13.5 and 1441 at P1 were differentially expressed, among 24,283 and 26,718 genes tested, respectively (Figure 4A,B). Gene counts after independent filtering and results based on FDR alone are given in Supplementary Note S2.

Given the role of GDNF–RET signalling in ureteric bud induction, we examined the expression of genes in this axis directly (Figure 4C). *Gdnf* and *Gfra1* were modestly but significantly lower in FVB/N at E13.5 (log_2_FC −0.48 and −0.52), and *Gfra1* remained lower at P1 (−0.56). The receptor *Ret*, however, was significantly higher in FVB/N at P1 (+0.49), and *Wnt9b* was also higher (+0.81). These findings do not indicate coordinated downregulation of the axis.

Consistent with this, no kidney gene set reached FDR < 0.1 at either stage. The strongest signal at both stages was negative enrichment of GO nephron morphogenesis, but the minimum adjusted *p* value was 0.22 at E13.5 and 0.35 at P1 (106 sets tested; E13.5 results are shown in Figure 4D).

### 2.5. No Between-Strain Difference Was Detected in Estimated Relative Cell Composition

**Figure 5:**
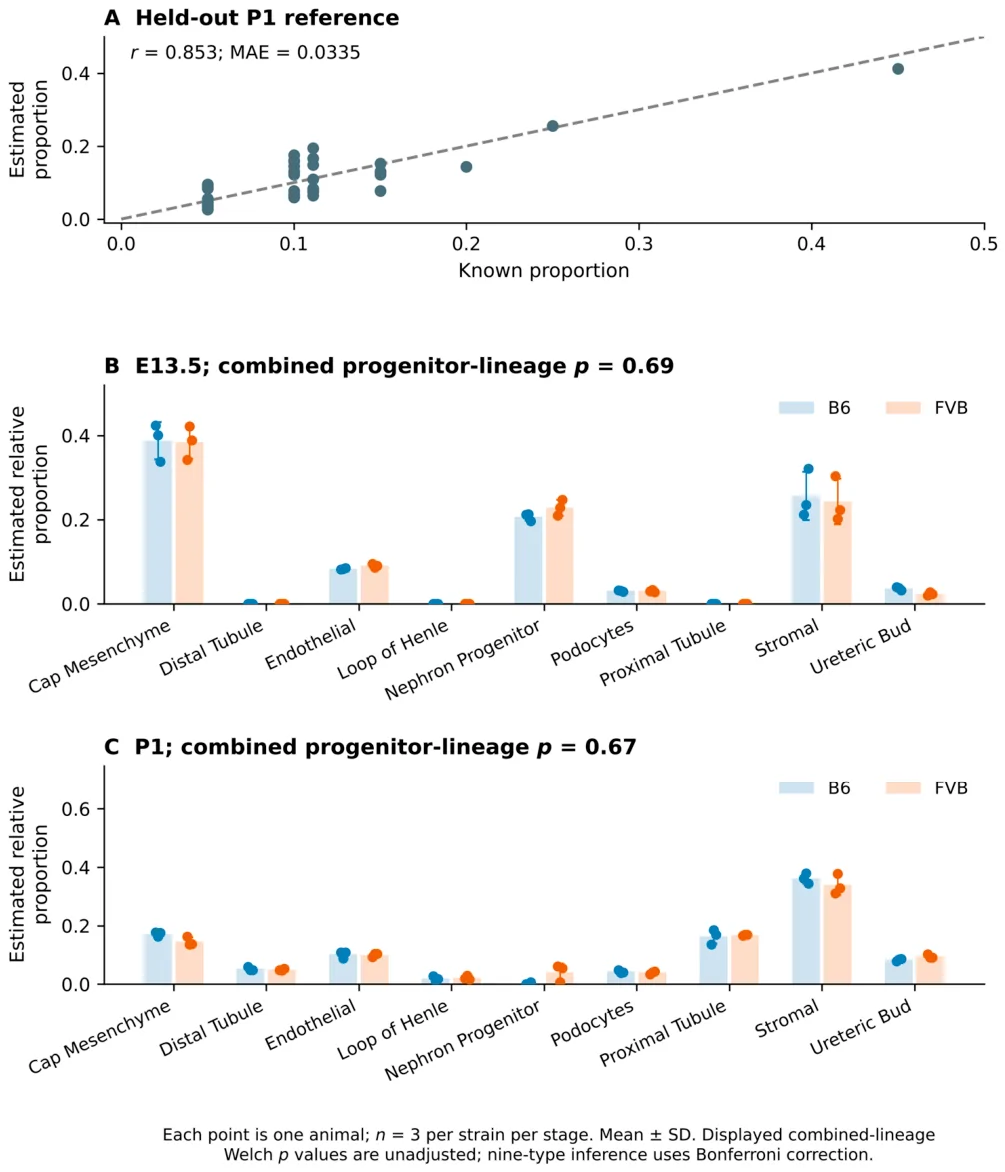
Relative cell-composition estimates using the P1 reference. (**A**) Known versus estimated proportions in four held-out pseudo-bulk mixtures from the same reference library (r = 0.853; MAE = 0.0335); the dashed line indicates identity (*y* = *x*). (**B**,**C**) Individual bulk-sample estimates at E13.5 and P1, respectively. Each point is one animal (*n* = 3 males per strain per stage); bars and error bars show mean ± SD; blue, C57BL/6J; orange, FVB/N. Welch *p* values for the combined cap-mesenchyme and nephron-progenitor lineage are unadjusted (0.69 and 0.67). Comparisons across cell types use Bonferroni correction across nine types within each stage. Estimates are reference-dependent and do not establish absolute cell numbers or equivalence. Embryonic-reference sensitivity is shown in Supplementary Figure S3. B6, C57BL/6J; FVB, FVB/N.

We estimated relative cell composition to assess whether it could contribute to differences in bulk gene expression. Evaluation using held-out cells from the P1 reference is shown in Figure 5A. Using the P1 reference, the combined cap-mesenchyme and nephron-progenitor estimate was approximately 60% at E13.5 and 18% at P1. Individual estimates are shown in Figure 5B,C.

The smallest unadjusted *p* value was 0.019 for ureteric bud at E13.5; it did not pass Bonferroni correction across the nine cell types within that stage (adjusted *p* = 0.17). The estimated proportion of the combined lineage did not differ significantly between strains (unadjusted *p* = 0.69 at E13.5 and 0.67 at P1).

An E14.5 whole-kidney reference comprising 4183 author-labelled cells [17] gave E13.5 progenitor estimates of 25.7% in C57BL/6J and 26.3% in FVB/N (difference +0.6 percentage points; 95% CI, −10.1 to +11.4; unadjusted Welch *p* = 0.88; Supplementary Figure S3). No between-strain comparison was significant after correction across the 11 reference populations (minimum adjusted *p* = 0.169). The P1 reference gave corresponding combined-lineage estimates of 59.4% and 61.3%. Absolute estimates were reference-dependent; nonsignificant contrasts do not establish equivalence or equal absolute cell numbers.

### 2.6. Exploratory Candidate Prioritisation

**Figure 6:**
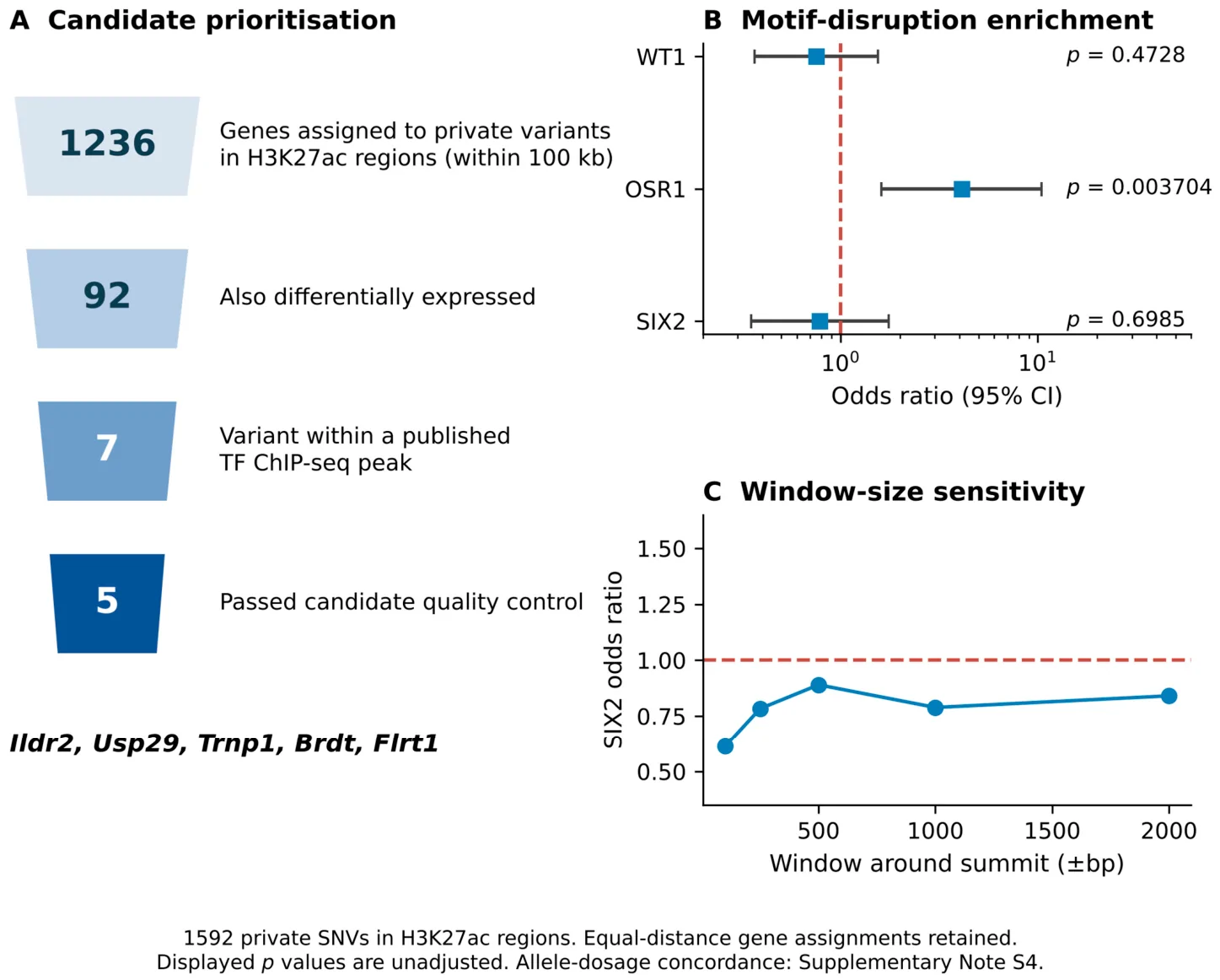
Candidate prioritisation and motif-disruption overlap. (**A**) Gene-level filtering: assignment of an FVB private variant in an H3K27ac region within 100 kb (1236), differential expression (92), published transcription-factor peak overlap (7), and quality control (5). Darker shading indicates later filtering steps. Equal-distance assignments were retained. (**B**) Odds ratios and 95% CIs for motif-disruption calls versus ChIP-seq overlap among 1592 private SNVs in H3K27ac regions; displayed *p* values are unadjusted; squares show odds ratios, and the dashed red line marks an odds ratio of 1. (**C**) SIX2 window-size sensitivity (odds ratio by window half-width around the peak summit; the dashed red line marks an odds ratio of 1). The OSR1 result used 19 in-peak variants and remained significant after correction across three factors.

Sequential integration of variant, chromatin, expression and transcription-factor occupancy data retained 1236 genes with assigned variants, then 92 differentially expressed genes, then 7 genes with an assigned variant overlapping a published transcription-factor peak, and finally 5 genes after quality control (*Ildr2*, *Usp29*, *Trnp1*, *Brdt* and *Flrt1*; Figure 6A; Table 2). None of these genes was included in the curated kidney-development or CAKUT lists.

Nine of the 15 unique regulatory variants have ambiguous assignments to overlapping genes: the eight *Flrt1* variants also map to *Macrod1*, and the *Brdt* variant to *Gm71444*. These assignments are based on genomic position and do not establish regulatory targets. The candidate variants were not individually confirmed in the experimental FVB/NJcl animals; their presence is therefore inferred from public FVB/NJ calls.

Motif-disruption calls were not enriched in SIX2 or WT1 peaks (odds ratios 0.78 and 0.75; raw *p* = 0.70 and 0.47). The SIX2 results were similar across the summit windows examined (Figure 6B,C). OSR1 enrichment was detected (odds ratio 4.10; raw *p* = 0.0037; adjusted *p* = 0.011 across three factors), based on 19 in-peak variants.

### 2.7. Unrestricted Exploratory Gene-Set Analysis Identifies Two Expression Programmes at P1

**Figure 7:**
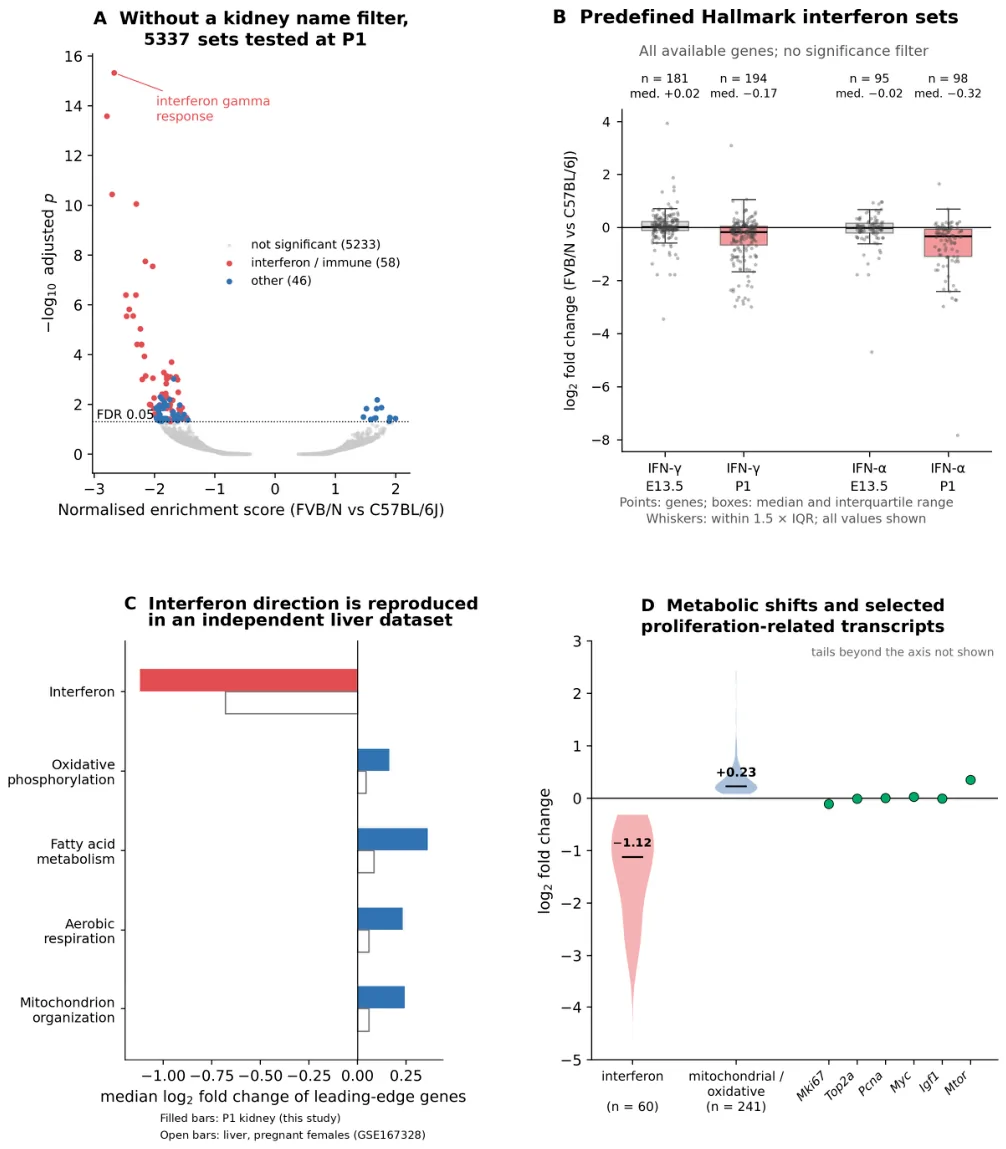
Unrestricted exploratory gene-set analysis. (**A**) P1 results for 5337 eligible sets after applying 15–500-gene size limits. Ranking was performed after excluding the 164 genes whose counts depended strongly on the reference genome (Section 4). Benjamini–Hochberg correction was applied jointly across collections within each stage. Of 104 sets with FDR < 0.05, 58 were interferon-/immune-related, and 46 were not; 5233 were not significant. Red, interferon-/immune-related sets; blue, other significant sets; grey, nonsignificant sets; the dotted horizontal line marks FDR = 0.05. (**B**) Gene-level log_2_FC for all analysed members of the predefined HALLMARK interferon-γ and interferon-α response sets. Gene counts at E13.5/P1 are 181/194 (γ) and 95/98 (α); membership differs from that used in panel A by four or five genes (Supplementary Note S2); corresponding medians are +0.02/−0.17 and −0.02/−0.32. Dots represent genes; boxes show the interquartile range, centre lines the median, and whiskers extend to the most extreme observations within 1.5 interquartile ranges. All points are shown. Red boxes denote P1 and grey boxes E13.5; the horizontal line marks log_2_FC = 0. No stage × strain interaction was tested. (**C**) Median log_2_FC of programme leading-edge genes in P1 kidney and liver from GSE167328: vehicle-treated pregnant females, mated at 6–8 weeks and sampled at gestation day 11, *n* = 3 per strain. Filled and open bars denote kidney and liver. Red and blue fills denote interferon-related and metabolic programmes, respectively; the vertical line marks zero. Concordant enrichment was observed for interferon-related sets; differences between the datasets preclude a formal tissue-specificity claim. (**D**) Leading-edge log_2_FC distributions and selected proliferation/growth-marker transcripts; bars indicate medians; red and blue violins show interferon and mitochondrial/oxidative leading-edge genes, green points show the marker transcripts, and the horizontal line marks log_2_FC = 0. No significant difference was detected in the six marker transcripts (all FDR > 0.18), which does not establish equivalent proliferation.

Unrestricted exploratory enrichment tested 5277 eligible sets at E13.5 and 5337 at P1 after the analysis-specific 164-gene exclusion (Section 4). At P1, 104 sets reached FDR < 0.05 (minimum adjusted *p* = 4.8 × 10^−16^; Figure 7A); at E13.5, one set met this threshold (HALLMARK hypoxia, adjusted *p* = 0.007). Without the exclusion, 92 sets reached FDR < 0.05 at P1, while the highlighted interferon and metabolic sets remained significant in the same direction.

Fifty-eight of the 104 significant P1 sets were interferon- or immune-related, all negatively enriched in FVB/N (HALLMARK interferon-γ response, normalised enrichment score (NES) −2.67; interferon-α response, NES −2.79). Across all analysed members of these two predefined HALLMARK sets, median log_2_FC was +0.02 and −0.02 at E13.5, compared with −0.17 and −0.32 at P1 (Figure 7B). Thus, strong set-level enrichment was accompanied by moderate median changes and larger changes in a subset of genes, including *Irf7* (−2.32) and *Ifit3* (−2.74). A formal stage × strain interaction was not tested, so differences in significance between stages do not establish stage specificity. *Ptprc* showed no significant expression difference (log_2_FC +0.06, FDR = 0.97), but a single marker cannot exclude altered immune-cell abundance or composition. Individual P1 interferon-γ and interferon-α scores were lower in all three FVB/N samples than in all three C57BL/6J samples. Omitting any one sample and repeating normalisation retained the negative strain difference in all six checks (Supplementary Figure S2D–F).

In GSE167328 [18], liver from vehicle-treated pregnant C57BL/6J and FVB/NJ females (*n* = 3 per strain) showed negative enrichment of the same interferon-response sets in FVB/NJ (NES −2.29 to −2.55, all FDR < 1 × 10^−8^; Figure 7C). The direction of enrichment was consistent with that in P1 kidney, although tissue, sex, age, pregnancy status and processing differed between datasets.

The remaining 46 significant sets predominantly involved mitochondrial and oxidative metabolism and were positively enriched in FVB/N (oxidative phosphorylation, fatty acid metabolism, aerobic respiration and mitochondrion organisation, adjusted *p* 0.007 to 0.032). Unlike the interferon signature, this shift was not reproduced in the liver dataset. The shift was small and was not accompanied by detectable differences in the measured proliferation-related transcripts: the leading-edge genes had a median log_2_ fold change of +0.23, and *Mki67*, *Top2a*, *Pcna*, *Myc*, *Igf1* and *Mtor* showed no significant expression difference (all FDR > 0.18; Figure 7D). We therefore treat both expression programmes as exploratory. With GRCm39-only quantification, all four highlighted metabolic sets remained positively enriched, but three had adjusted *p* values between 0.05 and 0.1 (Supplementary Note S2). Mitochondrially encoded genes contributed no more than two leading-edge genes to any of the four highlighted metabolic sets; all four remained significant when these genes were excluded.

## 3. Discussion

### 3.1. Recovered Counts and Kidney Mass

FVB/N had lower recovered glomerular counts than all three comparator strains, both per kidney and per gram of kidney, but the difference from C57BL/6J was not resolved after linear or allometric kidney-weight adjustment. The count-per-gram ratio therefore does not establish a deficit independent of kidney mass. Proportional scaling was not supported: within strains, heavier kidneys were not associated with higher recovered glomerular counts (common allometric exponent −0.26; Section 2.1). Because the FVB/N and C57BL/6J kidney-weight ranges do not overlap, the adjusted contrast between these two strains depends on extrapolating this common slope. Differences from BALB/c and DBA/2 persisted in the primary linear and allometric GLS models, although the FVB/N–DBA/2 contrast was borderline in the equal-variance OLS ANCOVA sensitivity analysis (Holm-adjusted *p* = 0.060).

Calculated kidney mass per recovered glomerulus was 29.3 ± 7.4 µg in FVB/N, compared with 14.9–17.1 µg in the other strains. This reciprocal index is derived from the same measurements and is not an independent endpoint or a measure of functional single-nephron load. Long et al. previously reported fewer counted glomeruli and greater albuminuria in FVB/N than in B6 mice [13]; the present study adds a four-strain comparison, age-associated morphometry and developmental transcriptomics.

### 3.2. Age-Associated Glomerular Enlargement and Urinary Estimates

The larger cross-sectional increase in glomerular long-axis diameter in FVB/N is compatible with compensatory enlargement [5,6,7], but neither absolute nephron endowment nor single-nephron filtration was established. Greater kidney mass at 8 weeks preceded the observed enlargement in separate older groups. The tissue compartments responsible for kidney-mass differences were not measured.

Semiquantitative UACR estimates were higher in FVB/N at both ages after correction across the four pairwise comparisons. The within-FVB/N age comparison was not significant after correction, and the strain × age interaction for log-transformed UACR was not significant. Comparisons of these independent age groups establish neither individual trajectories nor a temporal progression from glomerular enlargement to albuminuria. Differences in albumin permeability, tubular handling or creatinine excretion remain possible.

### 3.3. Reference-Genome Effects and Developmental Interpretation

The similar magnitudes but opposite directions of the shifts in the forward and reverse controls support strain-matched quantification. However, these controls cannot fully distinguish residual technical effects from genuine cis-regulatory divergence. The reversal in the direction of E13.5 translation- and ribosome-related enrichment between reference genomes indicates that these signals are reference-dependent and should not be interpreted as evidence of altered translation in FVB/N (Supplementary Note S2).

Neither the analysis using the P1 reference nor the sensitivity analysis using the E14.5 reference detected a strain difference in estimated progenitor proportion at E13.5. Absolute estimates differed substantially: approximately 59–61% with the P1 reference and 26% with the E14.5 reference. The references differ in developmental stage, cell labels, sex, strain and platform. Agreement between these statistical conclusions does not validate the absolute estimates, establish equivalence or exclude differences in absolute cell number or cell state.

The GDNF–RET axis showed discordant rather than coordinated changes: *Gdnf* and *Gfra1* were lower in FVB/N at E13.5, whereas *Ret* and *Wnt9b* were higher at P1. No kidney-related gene set reached FDR < 0.1. The analyses did not identify a clear transcriptomic explanation for the phenotype at either E13.5 or P1. They do not exclude changes in branching, progenitor-to-nephron conversion, absolute progenitor number or development after P1. Reference-allele preference alone does not establish that all normalised expression estimates are affected in the same direction.

### 3.4. Exploratory Regulatory Candidates

The five prioritised genes are candidates for independent testing. Nearest-gene assignment and overlap with a chromatin or transcription-factor peak do not demonstrate variant-to-gene regulation. The *Flrt1* variants also overlap *Macrod1*, and the *Brdt* variant overlaps *Gm71444*. Independent genotyping is required to confirm the candidate alleles. Numerous variants are confounded with strain, so the two-strain design cannot assign the phenotype to an individual locus.

Predicted motif-disrupting variants were not enriched within SIX2 or WT1 peaks. OSR1 enrichment remained significant after correction but was based on 19 variants within peaks. Allele-dosage concordance offered little independent support because 99.8% of background variants already met the criterion; it is reported descriptively in Supplementary Note S4. None of these analyses establishes a mechanism.

### 3.5. Exploratory Interferon and Metabolic Programmes

Lower P1 interferon-related expression was consistent across individual samples and after omission of each sample in turn. The independent liver data showed a concordant direction, suggesting a strain-associated pattern in these settings. Differences in tissue, sex, age, pregnancy status and processing preclude conclusions about a constitutive strain effect or tissue specificity. No stage × strain interaction was fitted. The modest mitochondrial/oxidative signature was not accompanied by significant changes in the selected proliferation or growth-factor markers. Neither programme has been established as an explanation for differences in kidney mass or recovered glomerular counts.

### 3.6. Limitations and Future Work

Recovered counts are extrapolated from isolated glomeruli and are not stereological measurements of total nephron endowment. The within-kidney CV describes aliquot precision, not the efficiency of digestion, filtration or recovery. Comparison with stereological estimates obtained by a different method [12] cannot establish recovery efficiency. Phenotypes at 8 weeks and developmental expression were measured in different animals, preventing animal-level expression–phenotype correlations. The small RNA-seq groups and reference-dependent deconvolution estimates limit the conclusions that can be drawn from nonsignificant results.

Measurements of glomerular count and kidney mass throughout nephrogenesis and postnatal growth are needed to establish when the phenotype emerges. Independent expression assays, genotyping and functional studies are required for the candidates. Crosses that introduce recombination could separate strain-confounded effects; allele-specific expression in reciprocal F1 hybrids could help distinguish cis and trans effects, with appropriate mapping controls [16,19,20].

The ageing analysis was cross-sectional, restricted to males and based on superficial cortical glomeruli. The UACR assay used a single-point BSA reference without independent confirmation of band identity or linearity. A longitudinal cohort including females, a validated mouse-albumin assay with a multipoint calibration curve, histological injury endpoints and direct filtration measurements would distinguish adaptive remodelling from injury. The present results do not establish a chronic kidney disease model or a causal link between recovered count, glomerular enlargement and albuminuria.

## 4. Materials and Methods

### 4.1. Ethics Statement

All animal procedures were conducted in accordance with the Act on Welfare and Management of Animals in Japan and the Kitasato University Regulations for the Care and Use of Laboratory Animals. The study is reported in accordance with the ARRIVE guidelines. The protocol was approved by the President of Kitasato University following review by the Institutional Animal Care and Use Committee (Approval ID: 25–018; approval date: 2 May 2025). A study protocol was prepared before the study began but was not registered in an external registry. For tissue collection from mice at 8 weeks of age and older, animals were deeply anaesthetised with an intraperitoneal injection of medetomidine (0.3 mg/kg), midazolam (4.0 mg/kg), and butorphanol (5.0 mg/kg) and euthanised by exsanguination via incision of the caudal vena cava. These doses were based on the three-drug combination described by Kawai et al. [21]. In adult mice, including pregnant dams, death was confirmed by cessation of respiration and heartbeat. Pregnant dams at E13.5 were euthanised by exsanguination via incision of the caudal vena cava under deep isoflurane anaesthesia. E13.5 embryos were euthanised by decapitation after removal from the uterus. P1 pups were anaesthetised with isoflurane and then euthanised by decapitation. Isoflurane was administered using an anaesthetic jar.

### 4.2. Animals

Male and female mice from four inbred strains—C57BL/6J (C57BL/6JJcl), FVB/N (FVB/NJcl), BALB/c (BALB/cByJJcl), and DBA/2 (DBA/2JJcl)—were used at 8 weeks (*n* = 5 per sex per strain; 40 animals in total). Mice were purchased from CLEA Japan, Inc. (Tokyo, Japan) at 8 weeks of age. No dedicated acclimatisation period was provided after arrival. Animals were maintained under specific pathogen-free conditions on a 12 h light/dark cycle at 22 ± 2 °C and 40–60% relative humidity, with free access to CE-2 chow (CLEA Japan, Inc.) and water.

Mice were housed in plastic Micro-Barrier cages (MBS7115HT; Allentown, Allentown, NJ, USA; floor area, 432 cm^2^), with a maximum of five mice per cage. Pulp bedding (Pulsoft; Oriental Yeast Co., Ltd., Tokyo, Japan) was used. No additional environmental enrichment was provided.

Animals were checked for health problems once weekly. No spontaneous deaths or health problems were observed during the housing period, including in the ageing cohort. No humane endpoints were predefined for this study.

Embryos and neonatal mice were obtained by breeding within the facility. The morning on which a vaginal plug was detected was designated embryonic day 0.5 (E0.5). For transcriptome analysis, kidneys were collected from male embryos at embryonic day 13.5 (E13.5) and from male pups at postnatal day 1 (P1), with *n* = 3 per strain per stage. Each sample comprised one animal, and the three animals within a group were taken from three different litters. The animals used for glomerular counting (8 weeks of age) and those used for RNA sequencing (E13.5 and P1) were separate cohorts; expression could therefore not be correlated with glomerular number at the individual-animal level.

An additional, independent cohort of male C57BL/6J and FVB/N mice was aged to 1 year and 1.5 years for phenotyping (see Ageing cohort and phenotyping below).

A total of 90 individual animals contributed to the analyses: 40 in the four-strain baseline cohort, 32 in the independent urine-sampling cohorts at 10 and 52 weeks, six in the 1.5-year groups, and 12 in the RNA-sequencing cohorts. Animals contributing to more than one measurement were counted only once.

Experimental unit, allocation, and sample size. The individual animal was the experimental unit in all analyses. Group membership was determined by genotype, so random allocation to strain was not applicable. The order of animal processing and measurement was randomised by shuffling animal identification numbers in both the baseline and ageing cohorts. The investigators collecting samples were aware of the animals’ strain and age. Sample sizes were not determined by a formal a priori power calculation; they were based on group sizes conventionally used for inbred-strain comparisons of this kind and on the number of animals available in the colony at each stage. No animals were excluded from the glomerular-count or ageing analyses after data collection.

### 4.3. Glomerular Counting

Kidneys were collected at 8 weeks of age, and body and kidney weights were recorded. The left kidney from each animal was used for glomerular counting, and each kidney was processed separately. The kidney weight used in Table 1 and count-to-weight calculations was the weight of this same left kidney. Kidneys were digested with 300 U/mL collagenase A (FUJIFILM Wako Pure Chemical Corporation, Osaka, Japan) at 37 °C for 45 min. Following digestion, complete liberation of the glomeruli from the surrounding tissue was confirmed microscopically. The digested suspension was passed through a 100-µm cell strainer, and the filtrate was subsequently passed through a 10-µm cell strainer (AS ONE Corporation, Osaka, Japan). Glomeruli retained on the 10-µm strainer were recovered and resuspended to a final volume of 5 mL. Immediately before each count, the suspension was vortexed for 10 s to ensure homogeneous dispersion. Five separate 50-µL aliquots were withdrawn from the same suspension and counted independently as technical replicates under a light microscope at ×40 magnification. Glomeruli were visually identified by their characteristic compact, approximately round capillary-tuft morphology. Only structurally intact glomeruli retaining a recognisable overall glomerular architecture were counted; fragmented or disrupted glomeruli, tubular fragments, and amorphous tissue debris were excluded. These criteria were established before glomerular counting. The investigator performing the counts was blinded to group allocation. No aliquot was recounted or excluded; all five aliquot counts from every kidney were retained. The mean of the five counts was multiplied by 100 to estimate the number of glomeruli recovered in the final suspension from each kidney.

Recovered glomerular count denotes the estimated number of isolated glomeruli, calculated by extrapolating the aliquot counts to the full suspension volume. It is reported per kidney and as recovered glomerular count per gram of kidney or body weight. These measurements do not establish recovery efficiency or absolute nephron endowment.

This method provides a comparison of recovered glomerular counts under a common protocol; equal recovery across strains has not been established. The value obtained for C57BL/6J differed substantially from the stereological estimate reported by Murawski et al. [12]. Because the two values were obtained using different measurement principles, they should not be compared directly.

### 4.4. Ageing Cohort and Phenotyping

Ageing phenotypes were assessed in male C57BL/6J and FVB/N mice from the same supplier and under the same housing conditions as the baseline cohort. Kidney and body weights were recorded at 8 weeks, 1 year and 1.5 years (*n* = 5, 5 and 3 per strain, respectively). The 8-week animals were the males in the four-strain baseline comparison; they were euthanised at 8 weeks, and no urine was collected from them. Glomerular long-axis diameter was measured at 10 weeks and 1 year (*n* = 5 per strain per age). UACR was measured at 10 and 52 weeks (C57BL/6J *n* = 5 and FVB/N *n* = 11 per age). The age groups comprised different animals, with no repeated urine sampling across ages.

The animals examined histologically also provided urine before euthanasia and formed a subset of the UACR groups. The remaining animals contributed urine only, without measurements of kidney weight or glomerular morphology. Group sizes differed because kidney measurements required terminal sampling, whereas urine was collected non-terminally from all available animals. More FVB/N males were available, and the additional animals were used for urine sampling because albuminuria was the most variable endpoint and was expected to be more pronounced in FVB/N.

Inferential analysis of glomerular counts was restricted to the common 8-week baseline. Differences in recovery or counting between cohorts preclude inference about glomerular gain or loss from these cross-sectional counts; ageing analyses therefore use kidney weight, morphometry and urinary estimates.

Histology and glomerular long-axis diameter. Kidneys were fixed in 10% neutral buffered formalin, paraffin-embedded, sectioned at 2 µm, and stained with periodic acid–Schiff (PAS). Sections were deparaffinised and rehydrated, oxidised in 0.5% periodic acid for 5 min, and rinsed in distilled water before incubation with Schiff reagent (FUJIFILM Wako Pure Chemical Corporation) for 30 min. Sections were then washed three times for 3 min each in 0.8% sodium bisulfite solution, washed under running water, counterstained with haematoxylin for 4 min, dehydrated, cleared, and mounted. Sections were imaged on an ECLIPSE Ti inverted microscope (Nikon Corporation, Tokyo, Japan). Images were calibrated at 0.120 µm per pixel. For each animal, the superficial renal cortex was scanned clockwise around the tissue section, and 20 consecutive glomeruli with a clearly visible urinary pole were selected. Juxtamedullary glomeruli were not included. Glomerular long-axis diameter was measured by an observer blinded to strain and age, and the per-animal mean was used as the unit of statistical analysis. Representative images in Figure 2A were cropped to a common 150 × 150 µm field centred on a glomerulus and are displayed at the same scale; no other adjustment was made.

Semiquantitative urinary albumin-to-creatinine ratio (UACR) estimation. Spot urine was collected by manual restraint and bladder compression. Samples were collected at the same time of day at each time point, and a single sample from each animal was analysed. Each urine sample was analysed once; therefore, a technical-replicate coefficient of variation was not calculated. Urine samples were mixed 1:1 (*v*/*v*) with SDS sample buffer. Twenty microlitres of the resulting two-fold diluted sample, equivalent to 10 µL of the original urine, was loaded per lane and separated by SDS-PAGE. A single reference lane containing 2.5 µg of bovine serum albumin (BSA) was included on the same gel. The sole prominent band detected in each urine lane was provisionally identified as urinary albumin with reference to the BSA control lane. No independent immunochemical confirmation of band identity was performed. Following background subtraction, albumin-band intensity was measured using ImageJ software (version 1.52a; National Institutes of Health, Bethesda, MD, USA) [22]. The amount of albumin in 10 µL of the original urine was estimated as 2.5 µg × (sample-band intensity/BSA-band intensity). This procedure used a single-point BSA reference rather than a multipoint calibration curve; analytical linearity was not independently established. The investigator performing the densitometric analysis was blinded to strain and age. Urinary creatinine was measured using the Creatinine (Urinary) Colorimetric Assay Kit (Cayman Chemical, Ann Arbor, MI, USA). A semiquantitative UACR estimate (mg/g creatinine) was calculated for each animal by normalising the BSA-equivalent albumin-band estimate to creatinine. All animals from which a urine sample of sufficient volume was obtained were included, and no sample was excluded after measurement. The urine-volume requirement for sample inclusion was established before analysis.

### 4.5. RNA Sequencing

Total RNA was extracted from kidneys dissected from E13.5 embryos and P1 pups using the NucleoSpin RNA Kit (Takara Bio, Kusatsu, Japan) according to the manufacturer’s instructions. RNA quantity, purity and integrity were assessed by the sequencing provider using a NanoDrop 2000 spectrophotometer, a Qubit RNA assay (both Thermo Fisher Scientific, Waltham, MA, USA) and an Agilent TapeStation system (RNA or High Sensitivity RNA ScreenTape; Agilent Technologies, Santa Clara, CA, USA). Only samples with an RNA integrity number equivalent (RINe) ≥ 7.0 were used; per-sample RINe values ranged from 8.3 to 10.0 (Supplementary Table S2). This RNA integrity threshold was established before analysis. Poly(A) mRNA was isolated using the NEBNext Poly(A) mRNA Magnetic Isolation Module, and strand-specific libraries were prepared using the NEBNext Ultra II Directional RNA Library Prep Kit (New England Biolabs, Ipswich, MA, USA). Library preparation and sequencing were performed by GENEWIZ/Azenta Life Sciences (Tokyo, Japan). Libraries were sequenced on a DNBSEQ-G400 instrument (MGI Tech, Shenzhen, China) to generate 150-bp paired-end reads. Per-sample sequencing output and quality statistics are reported in Supplementary Table S2.

Library type was assessed empirically with salmon v2.4.1 (--libType A), which inferred ISR (inward, stranded, reverse) for all twelve libraries, with a compatible-fragment ratio of 1.00. Quantification therefore used featureCounts v2.0.3 with -s 2.

Reads were trimmed and quality-filtered with fastp v1.3.6 [23] using default settings. Sample identity was verified before any downstream analysis: sex was confirmed from Y-linked transcripts (*Ddx3y*, *Eif2s3y*, *Uty*, *Kdm5d*) and *Xist*, and strain labels were confirmed by quantifying the fraction of FVB alleles at FVB-specific variant sites.

### 4.6. Extraction of FVB Private Variants and Personalised Genome Construction

FVB/N private variants were taken from Mouse Genomes Project release REL2021 (52 strains) [24,25]. Multiallelic records were decomposed into one record per alternate allele using bcftools (v1.21) norm -m -any [26]. Alternate-allele counts were recomputed with bcftools +fill-tags across all 52 strains before sample subsetting. Records were retained when FVB/NJ carried the alternate allele and its allele count was at most two. The FILTER=PASS restriction was applied to SNVs; no analogous restriction was imposed on the indel file, which carried no FILTER values in this release. The FVB/NJ sample was extracted only after filtering. This yielded 39,527 SNVs and 10,912 indels (50,439 total).

Because GRCm39 was derived from C57BL/6J and a matched call set for the C57BL/6JJcl animals was not available, C57BL/6J was treated as homozygous reference at the analysed sites. This design cannot detect C57BL/6J- or C57BL/6JJcl-specific variants. The MGP entry C57BL_6NJ represents a different substrain and was not used as a proxy.

The documented genome-construction workflow used variants carried by FVB/NJ independently of the private-variant filter used for candidate screening. SNVs were selected with FILTER=PASS, whereas no site-level FILTER restriction was applied to indels. After chromosome-name harmonisation, the SNV and indel VCFs were normalised against GRCm39 and decomposed with bcftools norm -m -any. The recorded inputs were FVB.snps.norm.vcf.gz for condition A and both FVB.snps.norm.vcf.gz and FVB.indels.norm.vcf.gz for condition B (Supplementary Table S1). Personalised FVB/N genomes were built with g2gtools v0.2.7 [27] (vcf2vci, patch, transform, convert) under two conditions: substitutions only (condition A) and substitutions together with indels (condition B). Alignments to the condition B genome were converted back to reference coordinates before quantification. Contigs that are declared in the VCI header but carry no variant records are absent from the coordinate mapping tree, and alignments on them are not converted; for FVB/NJ these were chrM, chrY and 39 unplaced scaffolds. A wrapper was therefore used that inserts an identity interval for each such contig, so that alignments on them are carried through unchanged (chrM 0 → 397,823 fragments and chrY 0 → 10,021 fragments in a representative sample). The wrapper used for reverse coordinate conversion, the patched bsam module, a unified diff against upstream g2gtools 0.2.7 and a minimal reproducible example are available at https://github.com/masakiwatanabe-labani/fvb-nephron-density (tag v1.0.2, commit d51e9107ec972275a5c039402b96a6370319442f; accessed on 5 November 2026), under g2gtools_patch/ (Supplementary Table S1). Condition A requires no coordinate conversion because substitutions alone do not alter reference coordinates. Genome construction used all nonreference variants carried by FVB/NJ in the selected input call sets (6,142,328 substitutions and 1,214,530 indels), rather than the private-variant subset. These counts describe the genome-construction inputs and are not the number of independent phenotype-associated loci.

Recorded software versions, reference-file identifiers, checksums and archived verification environments are provided in Supplementary Table S1. Missing original execution records are identified explicitly; archived verification sessions are dated separately from the original analysis.

### 4.7. Read Mapping and Quantification

Genome indices were built with STAR v2.7.10a [28] (--sjdbOverhang 149 --genomeSAindexNbases 14) from (i) the GRCm39 primary assembly with the GENCODE release M39 [29,30] comprehensive annotation on reference chromosomes, and (ii) each FVB personalised genome with its converted annotation. Paired reads were aligned with default STAR parameters, except --readFilesCommand zcat --outSAMtype BAM SortedByCoordinate. Fragments were assigned to genes with featureCounts v2.0.3 (Subread) [31] using -p --countReadPairs -s 2 -t exon -g gene_id, that is, fragment-level counting with reverse-stranded assignment, matching the library type inferred above; multi-mapping and multi-overlapping reads were not counted. Reads in all three arms (reference, condition A and condition B) were aligned and counted using these settings. The recorded reference files are GRCm39.primary_assembly.genome.fa and gencode.vM39.annotation.gtf. Supplementary Table S1 gives their checksums, source URLs and acquisition dates where recorded; unavailable historical acquisition dates are reported as not recorded.

Variant density was the number of unique FVB/NJ variant positions (substitutions and indels) per kilobase of the annotated GENCODE M39 gene span, from gene start to end. Multiple variant records at the same position were counted once. For each gene, the forward shift was the change in FVB/N log_2_ expression when quantified against the personalised genome instead of GRCm39; because the B6 term is unchanged, this is also the change in strain log_2_FC. The reverse shift used C57BL/6J reads and the same personalised-minus-GRCm39 convention. Both directions used the same normalisation. Density–correction and signed/absolute density–expression correlations were evaluated separately, using genes with |uncorrected log_2_FC| > 0.1. The magnitudes of forward and reverse shifts were compared only for genes evaluable in both analyses, with genes carrying no within-gene FVB variant also summarised separately.

For differential expression, a hybrid count matrix was used: counts for C57BL/6J samples were taken from the GRCm39 arm and those for FVB/N samples from the condition B arm, so each strain was quantified against its matching genome.

Sample-level PCA used log_2_(median-ratio-normalised count + 1) values and the 500 most variable eligible genes across all 12 samples and separately within each stage. Size factors were estimated from genes with positive counts in all 12 libraries; genes were eligible for PCA if they had at least ten counts summed across the samples included in that analysis. The 164-gene exclusion was not applied (Supplementary Figure S2A–C).

Mitochondrial and Y-chromosome genes were retained throughout the final RNA-seq analyses because coordinate conversion preserves these alignments. Removing them changed the number of differentially expressed genes by five at E13.5 and three at P1, did not alter the significance of any enriched gene set, and left deconvolution estimates unchanged to five decimal places. The four highlighted P1 metabolic gene sets also remained significant after removing mitochondrially encoded genes from the enrichment analysis.

### 4.8. Differential Expression and Gene-Set Analysis

Differential expression was assessed with DESeq2 v1.38.3 [32], fitting ~ strain separately at each developmental stage to the hybrid count matrix. Genes were tested if they had at least ten counts summed across the six samples at that stage. *p* values were adjusted using the Benjamini–Hochberg correction, with DESeq2 independent filtering; genes with missing adjusted *p* values were not counted as significant. The main threshold was FDR < 0.1; highlighting in the volcano plots and candidate screening additionally required |log_2_FC| > 0.5. No stage × strain interaction was fitted, and comparisons of significance across stages were not interpreted as a formal interaction test. For a reference-genome sensitivity analysis, the DESeq2, kidney-focused and unrestricted enrichment procedures were repeated without modification, with counts for both strains taken from the GRCm39 arm; unrestricted enrichment was run with and without the 164-gene exclusion described below (Supplementary Note S2).

Kidney-focused gene-set enrichment was performed with fgsea v1.24.0 [33], using fgsea() with its multilevel algorithm (minSize = 3, maxSize = 500, eps = 0, sampleSize = 1001, random seed 42). At each stage, genes were ranked by the DESeq2 Wald statistic for FVB/N versus C57BL/6J. Four compartment-specific gene sets were compiled by the authors from canonical markers of the developing kidney [3,34,35,36,37]: nephron progenitor (*Six2*, *Cited1*, *Eya1*, *Meox1*, *Sall1*, *Wt1*, *Osr1*), ureteric bud (*Ret*, *Wnt11*, *Calb1*, *Gfra1*, *Gata3*, *Wnt9b*), differentiating nephron (*Lhx1*, *Pax8*, *Jag1*, *Hnf1b*, *Podxl*, *Nphs1*, *Nphs2*), and stroma (*Foxd1*, *Meis1*, *Pdgfrb*, *Tcf21*). Gene sets were obtained from MSigDB [38] using msigdbr v26.1.0 [39]; the retained software and environment records identify msigdbr 26.1.0 and babelgene 22.9. MSigDB sets were retained if their names matched the case-insensitive regular expression kidney|nephro|(?<!ad)renal|metanephr|ureter|glomerul|podocyte, giving 98 GO:BP and 4 Reactome sets (106 in total).

Unrestricted exploratory enrichment used the HALLMARK [40], C2 CP:REACTOME, C2 CP:KEGG_MEDICUS and C5 GO:BP collections from MSigDB 2026.1, without restricting gene-set names to kidney-related terms. Genes were ranked by the DESeq2 Wald statistic after excluding the reference-sensitive genes described below. Genes with missing Wald statistics or missing or empty symbols were omitted; for duplicate symbols, the first gene in gene-ID order was retained. Eligible sets contained 15–500 ranked genes; other fgsea settings were eps = 0, sampleSize = 1001 and seed 42. Benjamini–Hochberg correction was applied jointly across all eligible collections within each stage; FDR < 0.05 defined significant exploratory enrichment.

The 164-gene screen was applied to unrestricted exploratory enrichment to limit strong reference-dependent signals. C57BL/6J counts against GRCm39 and the SNV-plus-indel personalised genome were compared; genes with ≥60 fragment counts summed across both alignments and |log_2_ shift| > 1 were flagged, and the union of genes flagged at either stage was excluded at both stages. The exclusion was not applied to differential expression, kidney-focused enrichment, deconvolution or candidate prioritisation. None of the highlighted GDNF–RET genes, author-curated kidney markers, five prioritised candidates or deconvolution markers (510 P1-reference and 607 E14.5-reference markers) overlapped the exclusion list. Exclusion totals and sensitivity results are in Supplementary Note S2; marker lists and signature-reconstruction checks are in Supplementary Data S1.

For Figure 7B, all analysed members of the predefined HALLMARK interferon-γ and interferon-α response sets were plotted using membership retrieved with Hallmark alone. Membership differences from the enrichment analysis and sensitivity checks are detailed in Supplementary Note S2. For Figure 7D, unique P1 leading-edge genes were pooled within the interferon group (the two HALLMARK interferon-response sets) and the metabolic group (HALLMARK oxidative phosphorylation and fatty acid metabolism, Reactome aerobic respiration and respiratory electron transport, and GO mitochondrion organisation).

For P1 interferon scores, median-ratio size factors were re-estimated using the six P1 samples. The complete Hallmark gene memberships used in Figure 7B were held fixed (194 interferon-γ and 98 interferon-α genes). The mean gene-centred log_2_-normalised expression was calculated for each sample. Each sample was omitted in turn, normalisation was repeated with five samples, and the strain difference was recalculated. DESeq2 and fgsea were not rerun for these descriptive checks.

For the independent liver comparison, author-provided gene counts from GSE167328 were analysed for vehicle-treated C57BL/6J (GSM5101961–GSM5101963) and FVB/NJ (GSM5101988–GSM5101990), three females per strain. In the source study, females were mated at 6–8 weeks of age, and liver tissue was collected from pregnant animals on gestation day 11. The original pipeline [18] used Bowtie2 v2.3.2 alignment to strain-specific pseudogenomes, conversion to common mm9 coordinates and HTSeq-count v0.6.1. We analysed the supplied counts using DESeq2 v1.38.3 (~ strain), ranked genes by the Wald statistic, and tested the HALLMARK and Reactome collections with the same fgsea parameters as in the unrestricted kidney analysis. Benjamini–Hochberg correction was applied across all eligible liver gene sets from these two collections before programmes were selected for comparison.

### 4.9. Regulatory Annotation

Variants were annotated with Ensembl VEP v116 [41] (GRCm39 cache, release 116, --everything).

Public histone tracks from GSE124804 [34] were obtained from CD1 nephron progenitor preparations at E13.5 and P0, enriched by magnetic sorting and expanded for two passages in nephron progenitor expansion medium (GSM5993848, GSM5993850, GSM5993852 and GSM5993854). H3K27ac peaks from E13.5 and P0 were merged into one catalogue and applied to both RNA-seq stages; H3K4me1 tracks provided contextual chromatin information. The peaks were converted from mm10 to GRCm39 using CrossMap v0.7.3 [42] and the UCSC chain. Conversion checks are given in Supplementary Note S2 and Table S1.

Transcription-factor tracks were obtained from developing kidneys of E16.5 CFW mice in GSE90016, the ChIP-seq subseries of GSE90017 [36]: NPC-directed FLAG-tagged SIX2, HOXD11 and OSR1 (GSM2395741–GSM2395743), native WT1 ChIP from wild-type kidney tissue (GSM2395744), and native SIX2 ChIP (GSM2867764). The WT1 dataset was not derived from purified NPCs. Each track was evaluated separately after summit conversion to GRCm39, with overlap defined within ±250 bp of a summit; overlap with any qualifying track could retain a candidate. The catalogue was applied to both RNA-seq stages and did not represent stage- or strain-matched measurements of occupancy.

Motif disruption was predicted with motifbreakR v2.12.3 [43,44] (threshold 1 × 10^−3^, method = “ic”) using all *Mus musculus* motifs in MotifDb v1.40.0 whose gene symbol matched one of eight priority transcription factors: SIX2, WT1, PAX2, PAX8, HNF1B, OSR1, TCF21 and FOXD1. No curated motif was available for SALL1 or EYA1 in this database, so these factors could not be tested. The input was the allele-wise private-variant set restricted to H3K27ac regions (1592 SNVs); because that set is decomposed to one record per alternate allele, every scored alternate allele is one that FVB/NJ carries (checked: 0 of 1592 variants had a scored allele differing from the FVB/NJ allele). The analysis identified 10,818 predicted disruption events involving 1551 variants.

### 4.10. Candidate Prioritisation

In the recorded BEDTools implementation, gene intervals were derived from the GTF gene records and represented as chromosome, start − 1, end and gene ID in a sorted BED file. Variant-to-gene distances were calculated with bedtools closest -d against these gene spans rather than against transcription-start-site points (Supplementary Table S1). Variants in H3K27ac regions were assigned to the nearest gene or genes within 100 kb; all genes tied at the same minimum distance were retained. Candidates were then prioritised by intersecting four evidence layers with BEDTools v2.31.1 [45]: (1) assignment of an FVB private variant in an H3K27ac region; (2) differential expression at the stage with the smallest available adjusted *p* value (FDR < 0.1 and |log_2_FC| > 0.5); (3) overlap of the assigned variant with a published transcription-factor ChIP-seq peak; and (4) quality-control criteria comprising mean normalised fragment expression (DESeq2 baseMean) ≥25, an absolute change in the CPM-based log_2_FC between the uncorrected and condition B arms <0.2, complete separation of the strains at the selected stage, and DESeq2 |log_2_FC| < 5 to exclude likely annotation or mapping artefacts. The stage was selected before applying the FDR and fold-change thresholds, and the subsequent quality-control criteria were evaluated at that stage. The correction shift was calculated from log_2_ ratios of strain-mean counts per million (CPM); complete separation required non-overlapping sample-level CPM ranges between strains in the condition B count matrix. Gene counts after these successive steps were 1236, 92, 7 and 5.

Variants equidistant from multiple genes were assigned to all nearest genes. All 295 ties in the recorded assignment set involved overlapping gene annotations and had distance zero. Positional assignment does not identify the true regulatory target.

This is a prioritisation procedure, not a formal test; the family-wise error rate was not controlled across the final candidate list.

### 4.11. Cell-Composition Deconvolution

To assess cell composition as a source of bulk-expression differences, relative cell-type proportions were estimated by deconvolution using GSE94333 [37] (Drop-seq of P1 mouse kidney) as the reference. All 4853 cells in the nine deposited cell-type label files were retained, using the original authors’ quality control and labels without additional cell filtering. Cell-type-specific marker genes were selected from the signature subset (mean expression > 20 CPM in the target population and among the 60 most specific genes for that population), yielding 538 unique markers, of which 510 were present in the bulk expression data. Relative cell-type proportions were estimated by non-negative least squares (R package nnls v1.6) after per-gene scaling; absolute cell numbers per kidney were not estimated. The cap-mesenchyme and nephron-progenitor signatures were closely related and collinear, so their estimated proportions were also summed as a combined lineage. The source files, cell counts and mapping of the nine deposited labels are documented in Supplementary Table S1.

The method was evaluated using pseudo-bulk mixtures of known composition. Within each cell type, reference cells were randomly permuted and assigned alternately to signature and validation subsets, using seed 42. Four mixtures were generated from the held-out validation cells by sampling with replacement at fixed proportions, targeting 2000 cells per mixture (1998 in the equal-proportion mixture because of rounding). Performance was assessed by Pearson correlation (r = 0.853) and mean absolute error (0.0335). Mixture compositions and computational details are documented in Supplementary Table S1. Because both subsets originated from the same P1 reference library, this evaluation applies only within that reference and does not validate performance in E13.5 kidneys or across strains. Thresholds of r > 0.8 and mean absolute error < 0.05 were used only as descriptive benchmarks and were not prespecified.

The sensitivity analysis using an embryonic reference used processed counts and original cell labels from GSE158166 [17] (GSM4794634; E14.5 female CD1 whole kidney, mm10; 4183 cells and 11 labelled populations). No additional cell filtering was applied. Cells were randomly permuted within each population using NumPy PCG64 seed 42 and assigned alternately to signature and validation subsets. The same marker-specificity and per-gene scaling principles were used; 642 markers were selected and 607 matched gene symbols in the bulk data using case-insensitive matching. Four pseudo-bulk mixtures were sampled with replacement from held-out cells, targeting 2000 cells each. Full mixture compositions, realised cell totals and validation results are reported in Supplementary Table S1 and Supplementary Data S1. Cell-type proportions in the E13.5 bulk samples were estimated using this reference. Welch tests used Bonferroni correction across 11 populations. The embryonic progenitor label was compared descriptively with the summed P1-reference cap-mesenchyme and progenitor labels; the cell-type classifications are not identical. Metadata, marker lists, sampled mixtures and estimates are supplied in Supplementary Data S1.

BisqueRNA [46] was not used because the reference dataset contained only one pooled library and therefore did not provide the between-subject information required to estimate the standard Bisque transformation reliably.

### 4.12. Four-Strain Allele-Dosage Analysis

Four-strain allele-dosage concordance was evaluated descriptively using public reference-strain genotype proxies. Because private-variant selection induced near-universal background concordance, it was not used as independent validation of the candidates. The full criteria, caveats concerning substrains and the 48,401/48,510 background result are given in Supplementary Note S4.

### 4.13. Statistics

Statistical analyses were not blinded. RNA-seq analyses used R v4.2.2 and Python v3.11.2. Additional phenotype models, sample-level RNA checks, and embryonic-reference sensitivity analyses used Python v3.12.14, NumPy v2.3.5, SciPy v1.17.0, and pandas v2.2.3. Scripts, environments and input identifiers are supplied in Supplementary Data S1 and Table S1. The original genomics release and additional revision analyses are identified separately.

Values are presented as mean ± SD, with the animal as the biological unit. Analyses used per-animal mean glomerular long-axis diameter and recovered-count estimates. Within-kidney SD and CV were calculated from the five aliquots; log CV was analysed with strain and sex using HC3 heteroskedasticity-robust inference.

Primary count and count-per-gram comparisons used Gaussian generalised least squares (GLS), with sex retained as a covariate and separate residual variances for the four strains estimated by restricted maximum likelihood. An additive ANCOVA included strain, sex and kidney weight. A log–log sensitivity model included strain, sex and log kidney weight. Strain-by-sex and strain-by-weight interactions were assessed in separate expanded models. Proportionality was evaluated by testing whether the common log–log weight coefficient equalled one.

Pairwise contrasts were standardised to equal representation of both sexes; covariate-adjusted contrasts were evaluated at the overall mean kidney weight. Holm correction covered all six strain pairs within each model. Pointwise 95% CIs and Bonferroni simultaneous 95% CIs are supplied in Supplementary Data S1. GLS t and Wald F tests used residual degrees of freedom and are approximate because variance parameters were estimated. Sensitivity analyses included ML likelihood-ratio tests of interactions and OLS/HC3 models. Nonsignificant results were not interpreted as evidence of equivalence. The exploratory male FVB/N–BALB/c TOST [47], with post hoc margins, is reported in Supplementary Note S3.

Kidney weight and glomerular long-axis diameter were analysed by strain × age ANOVA. Kidney-weight pairwise contrasts used Tukey–Kramer adjustment across all six strain-by-age groups. Diameter comparisons used Welch tests with Holm correction across the two ages. UACR was log-transformed for the strain × age model. Pairwise UACR comparisons used two-sided Welch tests on untransformed values, with Holm correction applied to a single family containing two between-strain contrasts at fixed age and two age contrasts within strain. Welch tests on log-transformed values were used as sensitivity analyses. Age groups were independent.

For DESeq2, Benjamini–Hochberg correction was applied across genes. For enrichment, it was applied across the 106 kidney-focused sets within stage or across all eligible unrestricted collections together within stage. P1-reference deconvolution used Welch tests with Bonferroni correction across nine types within stage; the combined progenitor-lineage contrast was reported separately without adjustment. Embryonic-reference sensitivity used Bonferroni correction across its 11 labels. Motif-overlap associations used Fisher tests with correction across three factors. Allele concordance and within-reference pseudo-bulk benchmarks were descriptive.

## 5. Conclusions

FVB/N had lower recovered glomerular counts than the other three strains, both per kidney and per gram of kidney. Primary kidney-weight-adjusted GLS models supported differences from BALB/c and DBA/2, whereas the difference from C57BL/6J remained unresolved. Neither measure establishes absolute nephron endowment or a kidney-mass-independent deficit. Comparisons of independent male age groups showed greater glomerular enlargement and higher semiquantitative UACR estimates in FVB/N. These cross-sectional associations do not establish causality.

Developmental analyses at E13.5 and P1 identified no clear explanation for the phenotype. Deconvolution using either the P1 or E14.5 reference detected no strain difference in progenitor proportion at E13.5; estimates were reference-dependent. No coordinated downregulation of the GDNF–RET axis or significant enrichment of kidney-related gene sets was detected. Lower interferon-related expression at P1, a modest metabolic signature and the five prioritised genes remain exploratory findings. Positional assignments, genotype proxies from public strain datasets and chromatin overlap do not establish regulatory targets or causal variants. Developmental phenotyping, independent genotyping and expression validation, followed by genetic or functional testing, are needed.

## Figures and Tables

**Table 1:** Phenotypic summary of four inbred mouse strains at 8 weeks. Values are mean ± SD; *n* = 10 per strain (5 males and 5 females). Kidney weight is the weight of the same left kidney used for glomerular counting.

Measure	BALB/c	DBA/2	C57BL/6J	FVB/N
*n*	10	10	10	10
Body weight (g)	26.7 ± 2.4	20.6 ± 1.6	20.0 ± 2.8	24.6 ± 3.4
Left kidney weight (g)	0.189 ± 0.027	0.140 ± 0.027	0.105 ± 0.017	0.176 ± 0.033
Kidney/body weight (%)	0.705 ± 0.044	0.676 ± 0.100	0.527 ± 0.041	0.711 ± 0.052
Recovered glomerular count per kidney	12,548 ± 2506	8330 ± 758	7196 ± 778	6104 ± 614
Recovered glomerular count per gram of kidney	68,592 ± 20,488	61,860 ± 14,947	70,924 ± 18,727	36,071 ± 8622
Recovered glomerular count per g body weight	477 ± 120	407 ± 53	370 ± 84	253 ± 46
Kidney weight per recovered glomerulus (µg)	15.8 ± 5.0	17.1 ± 4.2	14.9 ± 3.4	29.3 ± 7.4

**Table 2:** Five exploratory candidate genes prioritised by allele-wise variant filtering, reverse-stranded fragment quantification, chromatin annotation and quality control. Fold changes are FVB/N versus C57BL/6J; coordinates refer to GRCm39.

Gene	Ensembl ID	Variant	Nearest Summit Factor (Dist)	E13.5 log_2_FC (FDR)	P1 log_2_FC (FDR)	Mean Count
*Ildr2*	ENSMUSG 00000040612.15	chr1:166110338	Wt1 (5 bp); peaks Osr1;Wt1	−0.624 (0.353)	−0.807 (0.00014)	508.0
*Usp29*	ENSMUSG 00000051527.12	chr7:6861786	Six2 (BioTagFLAG) (250 bp)	−0.046 (0.991)	−0.686 (0.012)	244.0
*Trnp1*	ENSMUSG 00000056596.10	chr4:133217557	Six2 (BioTagFLAG) (207 bp)	−0.99 (0.074)	−0.888 (0.234)	38.0
*Brdt*	ENSMUSG 00000029279.17	chr5:107507754	Wt1 (87 bp)	0.112 (0.991)	−0.687 (0.074)	144.0
*Flrt1*	ENSMUSG 00000047787.10	chr19:7098725	Wt1 (68 bp); peaks Osr1; Wt1; Hoxd11	+0.337 (0.316)	−0.693 (0.030)	293.0

FDR, false-discovery rate. Mean count denotes the DESeq2 base mean across the six samples at the stage with the smaller adjusted *p* value. One representative regulatory variant is listed per gene; the five genes have 15 unique regulatory variants in total. Nine variants also map to an overlapping annotation: all eight *Flrt1* variants to *Macrod1* and the *Brdt* variant to *Gm71444*. Nearest-gene assignment is positional and does not establish the target gene or cis-regulatory activity. The 295 equal-distance ties in the full assignment set were all at distance zero and were retained for every overlapping gene. All final candidate variants were assigned the MODIFIER annotation. The presence of candidate alleles in the experimental FVB/NJcl animals is inferred from public FVB/NJ calls; the candidate variants were not individually confirmed.

## Data Availability

Raw sequencing reads and gene-level count and TPM matrices are publicly available in GEO under accession GSE348802 (https://www.ncbi.nlm.nih.gov/geo/query/acc.cgi?acc=GSE348802; accessed on 9 November 2026). Original analysis code and available source data are archived at https://github.com/masakiwatanabe-labani/fvb-nephron-density (tag v1.0.2, commit d51e9107ec972275a5c039402b96a6370319442f; accessed on 9 November 2026). MANIFEST.tsv maps the archived analyses to scripts and outputs. Additional revision analyses, aliquot-level source data, reconstructed marker lists, and embryonic-reference sensitivity outputs are provided in Supplementary Data S1; these additional files are not contained in the cited v1.0.2 release. Public datasets, including the E14.5 reference GSE158166, are listed in Table S1.

## Supplementary Materials

The following supporting information can be downloaded at: https://www.mdpi.com/article/10.3390/ijms272110056/s1. References [34,35,42,47] are cited in the Supplementary Materials.

## Abbreviations

ANCOVA	analysis of covariance
ANOVA	analysis of variance
BAM	Binary Alignment/Map format
BSA	bovine serum albumin
CAKUT	congenital anomalies of the kidney and urinary tract
ChIP-seq	chromatin immunoprecipitation sequencing
CPM	counts per million
CV	coefficient of variation
E13.5	embryonic day 13.5
FDR	false discovery rate
GEO	Gene Expression Omnibus
GLS	generalised least squares
GO	Gene Ontology
GRCm38 (mm10)	Genome Reference Consortium mouse build 38
GRCm39	Genome Reference Consortium mouse build 39
GTF	Gene Transfer Format
H3K27ac	histone H3 lysine 27 acetylation
H3K4me1	histone H3 lysine 4 monomethylation
HC3	heteroskedasticity-consistent covariance estimator type 3
indel	insertion-deletion
log_2_FC	base-2 logarithm of fold change
MAE	mean absolute error
MGP	Mouse Genomes Project
MSigDB	Molecular Signatures Database
NES	normalised enrichment score
NPC	nephron progenitor cell
P0	postnatal day 0
P1	postnatal day 1
PAS	periodic acid-Schiff
Q30	Phred quality score of at least 30
REML	restricted maximum likelihood
RINe	RNA integrity number equivalent
RNA-seq	RNA sequencing
SDS-PAGE	sodium dodecyl sulfate-polyacrylamide gel electrophoresis
SNV	single-nucleotide variant
SPF	specific pathogen-free
TOST	two one-sided tests
TPM	transcripts per million
UACR	urinary albumin-to-creatinine ratio
VCF	variant call format
VCI	Variant Call Information
VEP	Variant Effect Predictor
