# Supplementary material for: Recovered Glomerular Counts in Relation to Kidney Mass and Developmental Transcriptomes in FVB/N Mice
Source: Int J Mol Sci. 2026 Nov 9;27(21):10056. doi: 10.3390/ijms272110056 (PMC13763732; doi:10.3390/ijms272110056)
Supplement: Supplementary file 1 [file ijms-27-10056-s001.zip › Supplementary_Data_S1/figures/SupplementaryFigureS3_embryonic_reference.pdf]

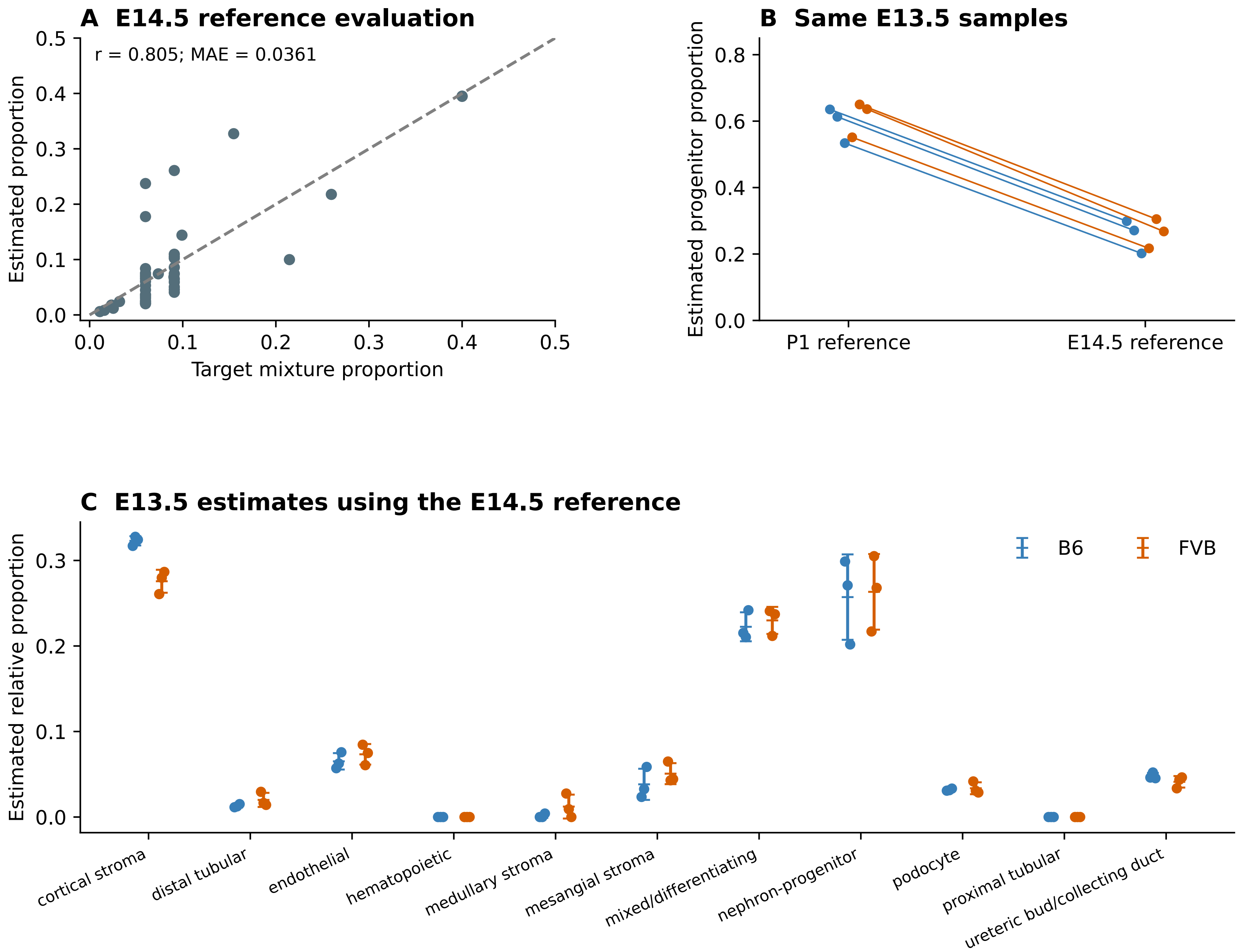

GSE158166: E14.5 female CD1 kidney; 4,183 labelled cells, 11 populations; 607 markers shared with bulk.
Lines in B connect estimates from the same sample. Reference cell partitions differ.
Welch strain tests with Bonferroni correction across 11 embryonic-reference populations.
