# Supplementary material for: Recovered Glomerular Counts in Relation to Kidney Mass and Developmental Transcriptomes in FVB/N Mice
Source: Int J Mol Sci. 2026 Nov 9;27(21):10056. doi: 10.3390/ijms272110056 (PMC13763732; doi:10.3390/ijms272110056)
Supplement: Supplementary file 1 [file ijms-27-10056-s001.zip › Supplementary_Information.pdf]

Supplementary information

Recovered glomerular counts in relation to kidney mass and developmental transcriptomes in FVB/N mice

Table S1 is supplied as a separate Excel workbook. Supplementary Note S1 on coordinate conversion is included in its Genome construction sheet. This document contains Tables S2–S5, Supplementary Notes S2–S4 and Figures S1–S3. Individual observations and reproducible analysis files are provided in Supplementary Data S1. Reference numbers correspond to the main manuscript.

Supplementary Table S2 Sequencing quality, RNA integrity and mapping statistics

Condition A incorporates SNVs only; condition B incorporates SNVs and indels. All libraries are male. These are the original three parts of the mapping table, moved from the main Results.

Sequencing quality and RNA integrity

Sample	Strain	Stage	Sex	Raw read pairs (M)	Clean read pairs (M)	Q30 (%)	RINe	28S/18S
B6135-1	B6	E13.5	M	30.6	30.6	96.6	8.3[ab]	2.3
B6135-2	B6	E13.5	M	28.7	28.7	96.5	10.0	1.9
B6135-3	B6	E13.5	M	30.1	30.1	96.8	10.0	2.1
B6P1-1	B6	P1	M	31.9	31.9	96.5	8.7	1.9
B6P1-2	B6	P1	M	28.7	28.7	96.7	8.9	2.1
B6P1-3	B6	P1	M	26.6	26.6	96.7	8.9	1.8
FVB135-1	FVB	E13.5	M	26.2	26.2	96.4	10.0[c]	2.0
FVB135-2	FVB	E13.5	M	30.5	30.5	95.8	8.3[ab]	2.7
FVB135-3	FVB	E13.5	M	33.2	33.2	96.6	8.8[a]	2.2
FVBP1-1	FVB	P1	M	28.0	28.0	96.8	8.3	1.6
FVBP1-2	FVB	P1	M	24.1	24.1	97.0	8.7	2.1
FVBP1-3	FVB	P1	M	29.3	29.3	96.8	8.6	2.0

Uniquely mapped fractions

Sample	Unique reference (%)	Unique A (%)	Unique B (%)
B6135-1	95.00	94.63	94.50
B6135-2	95.18	94.81	94.66
B6135-3	94.43	94.06	93.92
B6P1-1	94.04	93.78	93.61
B6P1-2	93.68	93.33	93.21
B6P1-3	93.28	92.90	92.77
FVB135-1	94.64	94.91	95.05
FVB135-2	94.43	94.72	94.86
FVB135-3	93.73	94.03	94.17
FVBP1-1	93.37	93.64	93.84
FVBP1-2	92.77	93.06	93.22
FVBP1-3	88.28	88.52	88.66

Fragment assignment

Sample	Assigned reference (%)	Assigned A (%)	Assigned B (%)
B6135-1	81.82	80.81	80.55
B6135-2	81.29	80.27	80.00
B6135-3	80.84	79.81	79.54
B6P1-1	73.52	72.79	72.51
B6P1-2	74.79	73.82	73.53
B6P1-3	74.15	73.11	72.81
FVB135-1	80.04	80.41	80.56
FVB135-2	79.55	79.99	80.14
FVB135-3	79.00	79.48	79.63
FVBP1-1	73.42	73.71	73.84
FVBP1-2	75.35	75.75	75.89
FVBP1-3	77.70	78.03	78.17

B6, C57BL/6J; FVB, FVB/N; M in the sex column, male; M in read-pair headings, millions of read pairs. Reference, GRCm39; A, SNVs only; B, SNVs plus indels. Assigned percentages refer to fragments counted with featureCounts -p --countReadPairs -s 2. Uniquely mapped percentages for conditions A and B are taken from STAR logs of alignments run with identical settings; logs of the final alignment were not retained (Table S1). Fragment-assignment percentages are from the featureCounts summaries of the final quantification. RINe (RNA integrity number equivalent) and 28S/18S area ratios were measured by the sequencing provider on an Agilent TapeStation before library preparation; all samples met the RINe ≥ 7.0 inclusion criterion. [a] Measured with High Sensitivity RNA ScreenTape. [b] The provider flagged the sample concentration as outside the recommended range. [c] The provider flagged the sample concentration as outside the functional range for RINe; the value is reported as measured.

Supplementary Table S3 Precision of the five aliquot measurements

Strain	Kidneys	Mean CV (%)	CV range (%)	Mean aliquot SD
B6	10	11.37	8.31–15.41	8.22
FVB	10	12.37	4.21–18.12	7.40
BALB	10	14.16	9.71–18.53	17.80
DBA	10	10.08	2.27–20.46	8.54

SD is calculated from the five raw counts per 50-µL aliquot; CV = 100 × SD / mean. All 200 aliquot observations were retained. No aliquot was recounted or excluded. Sex-adjusted log-CV analysis with HC3 inference did not detect an overall strain effect (p = 0.06630); no pairwise comparison was significant after Holm correction across six pairs. This evaluates aliquot precision, not biological recovery efficiency. All animal-level SDs and CVs are in source_data/animals_author_20261028.csv; raw aliquots are in source_data/aliquot_counts_200.csv.

Supplementary Table S4 Adjusted comparisons involving FVB/N

Model	Contrast	Estimate	Pointwise 95% CI	Holm *p*
Recovered count	FVB vs B6	−1092	−1646 to −538	0.000626
Recovered count	FVB vs BALB	−6444	−8001 to −4887	3.9×10^{-9}
Recovered count	FVB vs DBA	−2226	−2869 to −1583	1.73×10^{-7}

Model	Contrast	Estimate	Pointwise 95% CI	Holm *p*
Count per g kidney	FVB vs B6	−34,853	−43,763 to −25,943	1.46×10^{-8}
Count per g kidney	FVB vs BALB	−32,521	−42,511 to −22,531	6.15×10^{-7}
Count per g kidney	FVB vs DBA	−25,789	−35,646 to −15,932	2.5×10^{-5}
Count adjusted for kidney weight	FVB vs B6	−468	−1603 to 666	0.407
Count adjusted for kidney weight	FVB vs BALB	−6561	−8123 to −4998	3.45×10^{-9}
Count adjusted for kidney weight	FVB vs DBA	−1912	−2712 to −1111	7.93×10^{-5}
Adjusted count ratio	FVB vs B6	0.967	0.819 to 1.141	0.68
Adjusted count ratio	FVB vs BALB	0.483	0.422 to 0.554	8.27×10^{-12}
Adjusted count ratio	FVB vs DBA	0.776	0.695 to 0.866	0.000127

Sex is retained in each model and residual variances differ by strain. Differences are FVB minus the comparator; allometric estimates are FVB/comparator ratios. Holm correction covers all six strain pairs in each model, not only the three displayed here. Complete six-pair contrasts, Bonferroni simultaneous CIs, coefficients, interaction tests, ML sensitivities and independent optimisation checks are supplied in Supplementary Data S1. GLS t and Wald F inference is approximate because variances are estimated. B6 and FVB kidney-weight ranges do not overlap.

Supplementary Table S5 Reference-genome sensitivity of differential-expression and gene-set results

Values are strain-matched / GRCm39-only quantification. Strain-matched quantification is the primary analysis (C57BL/6J counted against GRCm39 and FVB/N against the SNV-plus-indel personalised genome); in the GRCm39-only analysis both strains were counted against GRCm39. DESeq2 and fgsea settings were identical.

Measure	E13.5	P1
Genes tested	24,283 / 24,341	26,718 / 26,765
DEGs (FDR < 0.1 and $\lvert \log_2 FC \rvert > 0.5$)	1186 / 1295	1441 / 1560
DEGs shared by both quantifications (Jaccard index)	1101 (0.80)	1367 (0.84)
DEGs called with one quantification only	85 / 194	74 / 193
One-quantification-only DEGs in the 164-gene list (%)	28.2 / 20.6	28.4 / 21.2
Correlation of $\log_2$FC (Pearson; Spearman)	0.939; 0.980	0.951; 0.982
Kidney-focused sets at FDR < 0.1 (minimum adjusted p)	0 (0.22) / 0 (0.25)	0 (0.35) / 0 (0.15)
HALLMARK interferon-γ response, adjusted p	—	4.8×10^{-16} / 1.0×10^{-16}
HALLMARK interferon-α response, adjusted p	—	2.6×10^{-14} / 4.1×10^{-15}
HALLMARK oxidative phosphorylation, adjusted p	—	0.015 / 0.073
HALLMARK fatty acid metabolism, adjusted p	—	0.014 / 0.070
Reactome aerobic respiration and respiratory electron transport, adjusted p	—	0.0066 / 0.015
GO mitochondrion organisation, adjusted p	—	0.032 / 0.068
Unrestricted sets at FDR < 0.05 with the 164-gene exclusion (shared)	1 / 8 (0)	—

DEG, differentially expressed gene. The 164-gene list contains genes whose C57BL/6J counts changed more than two-fold between GRCm39 and the personalised genome (Methods 4.8). Gene-set adjusted p values are from the unrestricted analysis with the 164-gene exclusion; the interferon sets were negatively and the metabolic sets positively enriched in FVB/N with both quantifications. At E13.5, the single strain-matched set (HALLMARK hypoxia) was not significant with GRCm39-only quantification, and the eight GRCm39-only sets (ten without the exclusion) were all translation- or ribosome-related. All five prioritised candidates were differentially expressed at their selected stage with both quantifications. Comparison tables and scripts are supplied in Supplementary Data S1/reference_sensitivity/ and scripts/.

Supplementary Note S2 Mapping validation and expression testing

Because GRCm39 is derived from C57BL/6J, FVB/N reads are expected to align less efficiently than C57BL/6J reads. Mapping FVB/N reads to the SNV-plus-indel FVB/NJ pseudo-genome increased the uniquely mapped fraction in every FVB/N sample (+0.430 percentage points, SD 0.032; paired t-test $p = 4.6 \times 10^{-7}$; Supplementary Figure S1A,B). In the reverse-control mapping of C57BL/6J reads to the same pseudo-genome, the uniquely mapped fraction decreased in every sample (−0.490 points, SD 0.034; paired t-test $p = 3.5 \times 10^{-7}$). The between-strain mapping rates with GRCm39 did not differ significantly (94.27% versus 92.87%; p = 0.195). One FVB/N sample, FVBP1-3, had a low mapping rate (88.28%) that remained low with the pseudo-genome (88.66%), supporting a sample-specific cause; its RNA integrity was high (RINe 8.6; Supplementary Table S2), and the sample was retained.

Changes after mapping correction were assessed in genes with large uncorrected between-strain expression differences; this selection can include both biological differences and technical mapping effects. Among genes with $|\text{uncorrected } \log_2FC| > 1$ (n = 387 at E13.5, n = 563 at P1), mean $|\log_2FC|$ fell from 1.99 to 1.71 (condition A) and 1.67 (condition B) at E13.5, and from 1.88 to 1.71 and 1.69 at P1 (Supplementary Figure S1C). Genome-wide mean $|\log_2FC|$ values were essentially unchanged (0.198 and 0.264 before correction). Estimates from the SNV-only and SNV-plus-indel genomes agreed closely (Pearson r = 0.989 at E13.5 and 0.992 at P1; Supplementary Figure S1E).

Correction magnitude was weakly positively associated with FVB variant density (Spearman ρ = 0.076 and 0.054 at E13.5, and 0.060 and 0.036 at P1, for conditions A and B; genes with $|\text{uncorrected } \log_2FC| > 0.1$, n = 7377 and 11,124; Supplementary Figure S1D). At E13.5, condition A quartile means were 0.016, 0.011, 0.022 and 0.041. These statistics describe variant density versus the magnitude of correction, not residual density–expression association. In a separate comparison, correlations between density and signed strain $\log_2FC$ changed from −0.074 to −0.052 and −0.037 at E13.5 and from −0.069 to −0.052 and −0.041 at P1. Correlations with absolute strain $\log_2FC$ changed only modestly, from 0.256 to 0.242 and 0.241 at E13.5 and from 0.204 to 0.196 and 0.198 at P1. The latter can reflect both technical effects and genuine cis-regulatory divergence; these components cannot be separated in this design.

In the reverse control, C57BL/6J reads were counted on the FVB personalised genomes. When forward and reverse shifts were compared using the same genes and metric (n = 7028 at E13.5 and 10,720 at P1), mean absolute shifts were similar in magnitude: 0.021 forward and 0.022 reverse at E13.5 under condition A, with forward/reverse ratios of 0.92–1.01 across stages and conditions. Per-gene shifts were oppositely directed (Spearman ρ = −0.55 to −0.62; Supplementary Figure S1F). The opposite shifts support quantification against strain-matched genomes, although they do not rule out residual technical bias. Shifts also occurred in genes carrying no FVB variant (reported means, 0.020 forward and 0.031 reverse), showing that genome-wide changes cannot be attributed solely to variants within the affected genes.

Of 24,283 tested genes at E13.5 and 26,718 at P1, 21,415 and 24,121 retained adjusted p values after independent filtering. FDR < 0.1 alone identified 1796 and 1640 genes; requiring $|\log_2FC| > 0.5$ as well identified 1186 and 1441. The main text and Figure 4 report the joint criterion.

Reference-genome sensitivity. To assess whether strain-matched quantification changes the differential-expression results or biological conclusions, the DESeq2, kidney-focused and unrestricted gene-set analyses

were repeated with both strains counted against GRCm39 (Supplementary Table S5). Of the 1186 (E13.5) and 1441 (P1) DEGs called with strain-matched quantification, 1101 (93%) and 1367 (95%) were also called with GRCm39-only quantification, and $\log_2$FC estimates were highly correlated (Spearman $\rho = 0.98$ at both stages). Calls that differed between quantifications were concentrated among the 164 reference-sensitive genes (20.6–28.4% of one-quantification-only calls, compared with 0.51–0.58% of tested genes). The main conclusions were retained: GDNF–RET axis estimates were nearly unchanged (E13.5 Gdnf and Gfra1 lower, P1 Ret and Wnt9b higher in FVB/N), no kidney-focused set reached FDR < 0.1, all five prioritised candidates remained differentially expressed at their selected stage (as expected from the correction-shift criterion), and the P1 interferon-response sets remained strongly negatively enriched. The four highlighted P1 metabolic sets remained positively enriched, but three had adjusted p values between 0.05 and 0.1 with GRCm39-only quantification. At E13.5, GRCm39-only quantification yielded eight significant translation- or ribosome-related sets, negatively enriched in FVB/N (NES −1.9 to −2.1), that were positively signed but non-significant with strain-matched quantification (NES +1.0 to +1.3); ribosomal-protein genes formed 62–100% of their leading edges. These sets are regarded as reference-dependent.

Unrestricted enrichment sensitivity. The reference-sensitive screen flagged 150 genes at E13.5 and 139 at P1; their union contained 164 genes. Applying this union at both stages removed 120 tested genes at E13.5 and 118 at P1. After omitting missing statistics or symbols and collapsing duplicate symbols, 24,143 and 26,581 genes remained ranked, respectively. Of 10,082 loaded gene sets, 5277 at E13.5 and 5337 at P1 met the 15–500-gene size limits. Without the exclusion, 92 rather than 104 sets reached FDR < 0.05 at P1, including 49 rather than 58 interferon-/immune-related sets. The interferon-response and mitochondrial/oxidative sets used in Figure 7D remained significant, with changes in normalised enrichment score of at most 0.03.

Hallmark membership for Figure 7B. Membership was retrieved from Hallmark alone with msigdbr 26.1.0. It differed from the enrichment-analysis membership, obtained by retrieving collections sequentially within the same session, by five interferon-γ genes (*Epsti1*, *Isoc1*, *Lysmd2*, *Samd9l* and *Sp110*) and four interferon-α genes (*Epsti1*, *Samd9l*, *Sp110* and *Tmem140*). Using enrichment-analysis membership (176/189 analysed genes for interferon-γ and 91/94 for interferon-α at E13.5/P1) changed the medians and fractions of genes with FDR < 0.1 by at most 0.009.

Chromatin coordinate checks used the GSE124804 histone tracks described in Methods 4.9 [34,35]. The peaks, originally called on mm10 (GRCm38), were converted to GRCm39 with CrossMap [42] and the UCSC mm10ToMm39 chain file. Conversion succeeded for all peaks in all four tracks. A reciprocal lift (GRCm39 → mm10 → GRCm39) of the E13.5 H3K27ac track returned 99.82% of intervals (27,520/27,569) to their original coordinates. For this nuclear-chromatin overlap check, the 500 most highly expressed genes at E13.5 were considered and seven chrM genes were omitted, leaving 493 genes. This restriction defines the chromatin quality-control subset and is separate from any gene filtering in the RNA-seq analyses. The proportion whose transcription start site ±2 kb overlapped the merged E13.5 and P0 H3K27ac peaks increased from 31.0% (153/493) before conversion to 96.3% (475/493) after conversion; the mean overlap across three shuffled controls was 13.0% (64/493).

Supplementary Note S3 Exploratory comparison of male kidney weights

The FVB/N–BALB/c male TOST [47] used a post hoc margin of ±20% of the BALB/c mean (n = 5 per strain, pooled df = 8). Kidney weight: FVB minus BALB/c = −0.0106 g, 90% CI −0.0302 to +0.0090 g, margin ±0.0425 g, p = 0.0081. Kidney-to-body-weight ratio: difference +0.0029 percentage points, 90% CI −0.0382 to +0.0440, margin ±0.1478 points, p = 0.000088. Mean recovered counts are 5888 and 11,264 in FVB/N and BALB/c males, respectively (1.91-fold difference). This selected comparison is descriptive, does not validate proportional scaling and is not a substitute for the primary sex-adjusted covariate models.

Supplementary Note S4 Descriptive four-strain allele concordance

Allele dosage was compared using reference-strain genotype profiles (C57BL/6J, BALB/cJ, DBA/2J and FVB/NJ) as proxies for the four phenotyped strain backgrounds. The profiles were ordered according to recovered glomerular count per gram of kidney weight in the phenotyped animals (C57BL/6J > BALB/c > DBA/2 > FVB/N). These profiles were not obtained by genotyping the experimental animals; in particular, the BALB/c animals were BALB/cByJJcl rather than BALB/cJ. Variant records were matched by chromosome, position, reference allele and alternate allele. From the 50,439 allele-wise private variants, 48,510 had complete genotype information for this analysis. A variant was called concordant when dosage was non-decreasing as recovered count per gram of kidney decreased, with at least one real increase; the C57BL/6J dosage was fixed at 0. Of these 48,510 variants, 48,401 met the criterion (99.775%, reported as 99.8%). All 15 unique candidate regulatory variants met the concordance criterion (15/15, 100%). Because the selection rule induces near-universal background concordance and the variants are not independent biological replicates, these proportions are treated descriptively; a binomial p value is not used as evidence of candidate validity.

The revised count data, based on all five aliquots, retain the ordering C57BL/6J > BALB/c > DBA/2 > FVB/N. Because the rank used in this rule is unchanged, the archived allele-concordance values are unchanged. This result is omitted from the main candidate figure and is not presented as independent evidence for the five candidates. The ambiguous *Flrt1*/*Macrod1* and *Brdt*/*Gm71444* assignments are retained explicitly in main Table 2.

Supplementary Figure S1 Detailed mapping validation

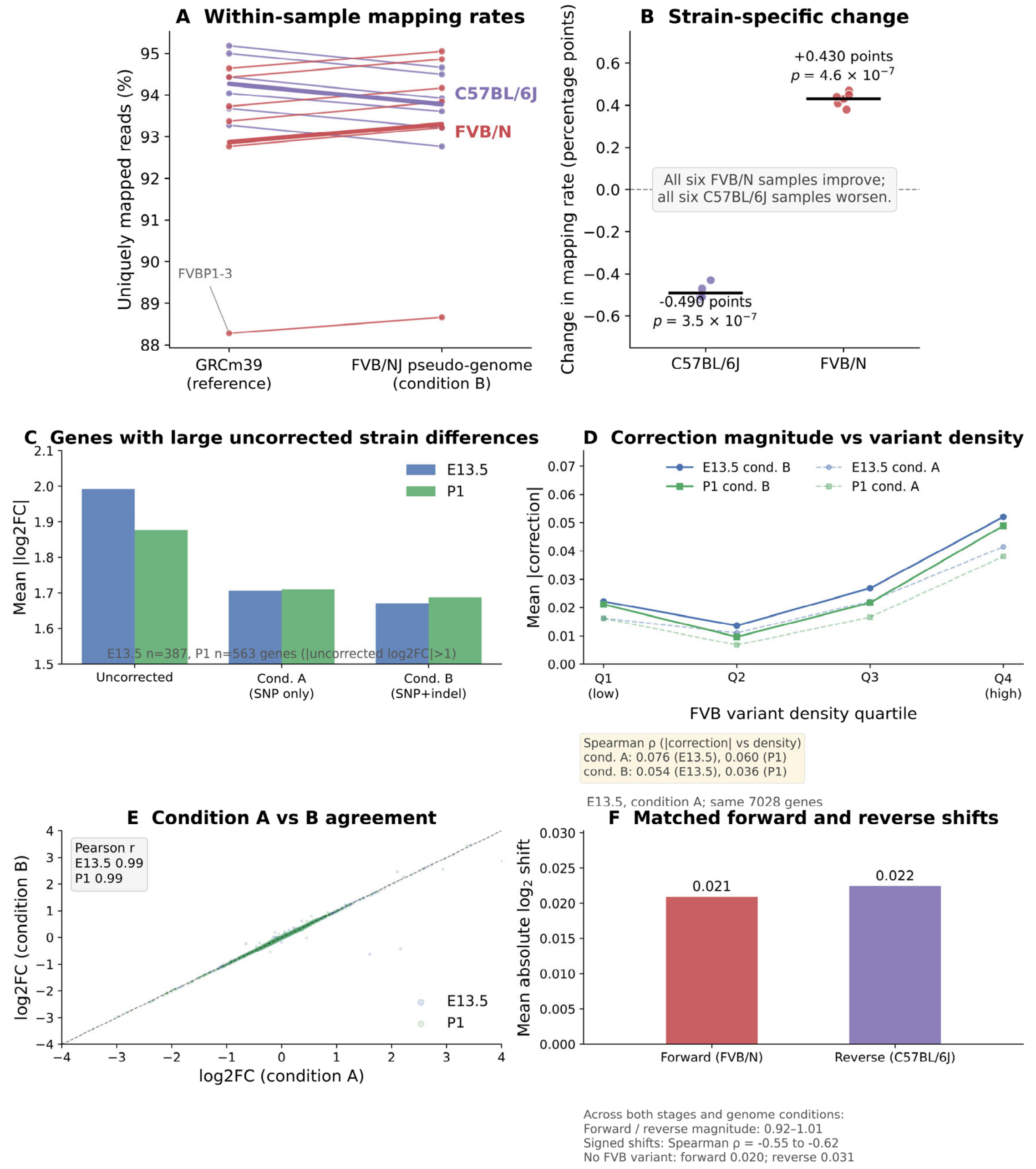

(A,B) Within-sample mapping fractions and strain-specific mapping changes. (C) Mean absolute strain $\log_2$FC in genes with large uncorrected differences. (D) Correction magnitude by variant-density quartile. (E) Agreement between SNV-only and SNV-plus-indel effect estimates. (F) Matched forward and reverse controls: mean absolute shifts 0.021 and 0.022 for E13.5 condition A, respectively; forward/reverse ratios 0.92–1.01 across stages and conditions. Full quantitative definitions, sample-retention decisions and residual-bias limitations are in Supplementary Note S2. The archived mapping-validation image is retained.

Supplementary Figure S2 Sample-level RNA checks

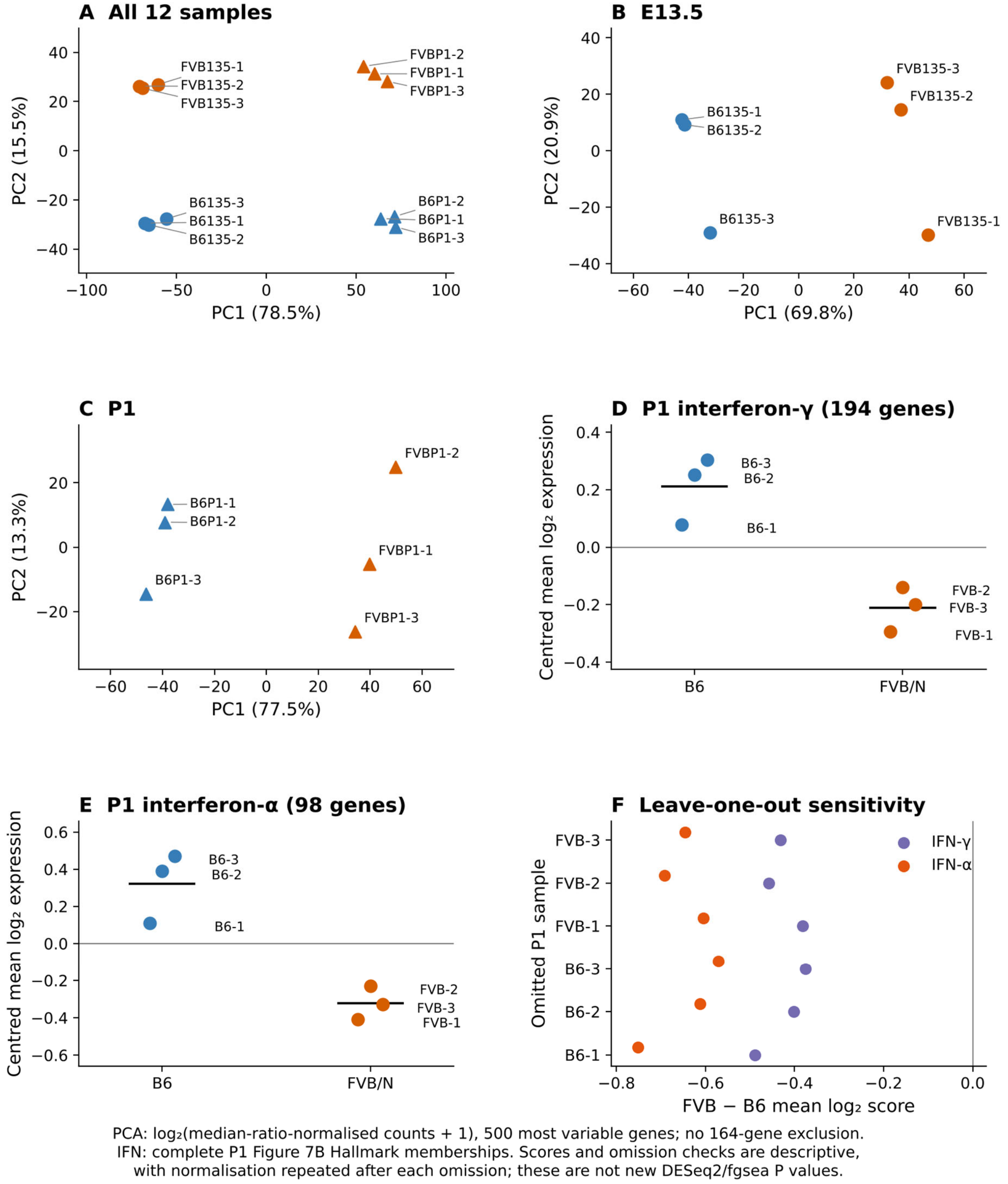

(A–C) PCA across all 12 samples, E13.5 only and P1 only, using $\log_2$(median-ratio-normalised counts + 1) and the 500 most variable eligible genes. The 164-gene exclusion was not applied. (D,E) Individual P1 gene-centred mean $\log_2$ expression scores for all 194 interferon-γ and 98 interferon-α members used in Figure 7B. Each point is one animal. (F) FVB-minus-B6 score differences after omission of each P1 sample and re-estimation of normalisation factors. Every omission retained the negative direction. These are descriptive score checks and do not constitute new DESeq2 or fgsea significance tests.

Supplementary Figure S3 Embryonic-reference sensitivity

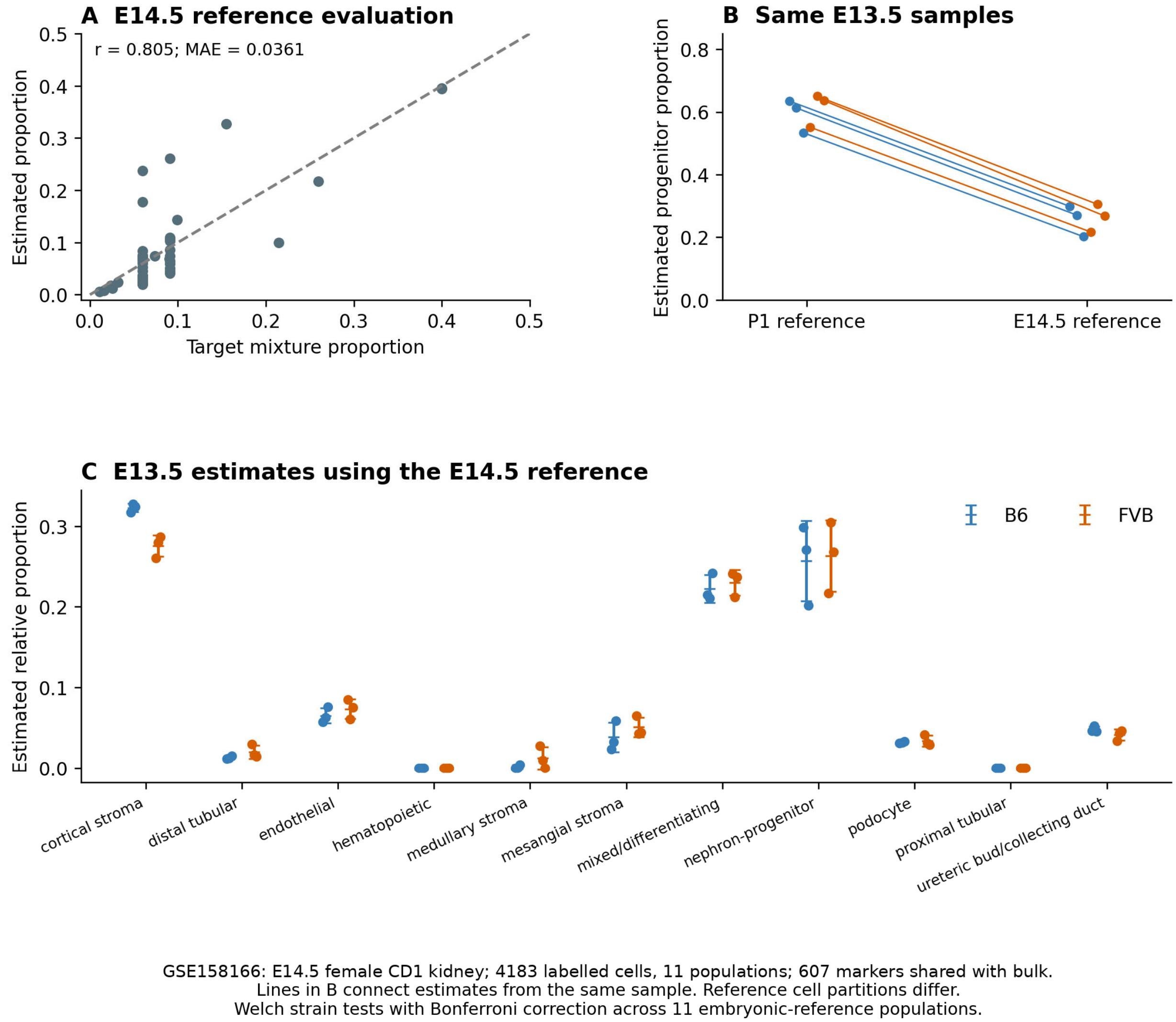

(A) Four held-out mixtures from the E14.5 reference (GSE158166; r = 0.805, MAE = 0.0361), using the regenerated benchmark documented in Table S1. The x-axis shows target mixture proportions. (B) Progenitor estimates for the same six E13.5 bulk samples using P1 and E14.5 references. Lines connect estimates from the same sample. The P1 values combine cap-mesenchyme and nephron-progenitor labels; the E14.5 values use its original progenitor label. These partitions are not identical. (C) Individual estimates for all 11 E14.5-reference populations; means and SD are shown. No comparison passed Bonferroni correction across 11 labels (minimum adjusted p = 0.169). The progenitor comparison gave unadjusted Welch p = 0.881. Within-reference mixture performance does not establish accuracy at E13.5. Absolute proportions depend strongly on reference stage and label composition.
